# Topologically inferring active miRNA‐mediated subpathways toward precise cancer classification by directed random walk

**DOI:** 10.1002/1878-0261.12563

**Published:** 2019-08-27

**Authors:** Ziyu Ning, Chenchen Feng, Chao Song, Wei Liu, Desi Shang, Meng Li, Qiuyu Wang, Jianmei Zhao, Yuejuan Liu, Jiaxin Chen, Xiaoyang Yu, Jian Zhang, Chunquan Li

**Affiliations:** ^1^ School of Medical Informatics Harbin Medical University Daqing China; ^2^ School of Pharmacology Harbin Medical University Daqing China; ^3^ Department of Mathematics Heilongjiang Institute of Technology Harbin China; ^4^ College of Bioinformatics Science and Technology Harbin Medical University China; ^5^ The Higher Educational Key Laboratory for Measuring & Control Technology and Instrumentations of Heilongjiang Province Harbin University of Science and Technology China

**Keywords:** cancer biomarker, classification, miRNA‐mediated subpathway, topological information

## Abstract

Accurate predictions of classification biomarkers and disease status are indispensable for clinical cancer diagnosis and research. However, the robustness of conventional gene biomarkers is limited by issues with reproducibility across different measurement platforms and cohorts of patients. In this study, we collected 4775 samples from 12 different cancer datasets, which contained 4636 TCGA samples and 139 GEO samples. A new method was developed to detect miRNA‐mediated subpathway activities by using directed random walk (miDRW). To calculate the activity of each miRNA‐mediated subpathway, we constructed a global directed pathway network (GDPN) with genes as nodes. We then identified miRNAs with expression levels which were strongly inversely correlated with differentially expressed target genes in the GDPN. Finally, each miRNA‐mediated subpathway activity was integrated with the topological information, differential levels of miRNAs and genes, expression levels of genes, and target relationships between miRNAs and genes. The results showed that the proposed method yielded a more robust and accurate overall performance compared with other existing pathway‐based, miRNA‐based, and gene‐based classification methods. The high‐frequency miRNA‐mediated subpathways are more reliable in classifying samples and for selecting therapeutic strategies.

**Abbreviations**
AKT2: AKT serine/threonine kinase 2
AUC: area under the curve
BRCA: breast invasive carcinoma
CAMs: cell adhesion molecules
GDPN: global directed pathway network
GEO: Gene Expression Omnibus
HF: high frequency
HIF: hypoxia‐inducible factor
HMDD: Human microRNA Disease Database
HNSC: head and neck squamous cell carcinoma
KEGG: Kyoto Encyclopedia of Genes and Genomes
KICH: kidney chromophobe carcinoma
KIRC: kidney clear cell carcinoma
KIRP: kidney papillary cell carcinoma
LIHC: liver hepatocellular carcinoma
LNCaP: lymph node carcinoma of prostate
LUSC: lung squamous cell carcinoma
miDRW: miRNA‐mediated subpathway activities using directed random walk
NOB1: NIN1 (RPN12) binding protein 1 homolog
PDGF‐β: platelet‐derived growth factor β chain
PD‐L1/CD274: programmed death‐ligand 1
PRAD: prostate adenocarcinoma
STAD: stomach adenocarcinoma
TCEB1: transcription elongation factor B polypeptide 1
TCGA: The Cancer Genome Atlas
TGF: transforming growth factor
THCA: thyroid carcinoma
UCEC: uterine corpus endometrioid carcinoma
VEGF: vascular endothelial growth factor

## 1 Introduction

MicroRNAs (miRNAs) are short, endogenous, noncoding RNAs that regulate post‐transcription by inhibiting the expression of target genes, thereby affecting the initiation, progression, and prognosis of cancers (Chen *et al*., 2012; Li *et al*., 2015; Luan *et al*., 2015; Rottiers and Naar, 2012; Tomasetti *et al*., 2014; Zhang *et al*., 2014). Many high‐throughput miRNA expression profiling studies have been performed with the aim to identify disease‐relevant miRNAs for clinical utility in diagnostic and prognostic applications (Bagnoli *et al*., 2016; Lin *et al*., 2015; Meiri *et al*., 2012; Xu *et al*., 2011). Moreover, a number of studies reported that the miRNAs were stable both in the body and in paraffin blocks, which provided better biomarkers of tumor classification (Baker, 2010; Iqbal *et al*., 2015; Lu *et al*., 2005; Matamala *et al*., 2015; Raponi *et al*., 2009; Zen and Zhang, 2012). Thus, some researchers proposed several methods to find miRNA biomarkers of the cancers, such as miRNA instance‐based approaches and miRNA feature‐based approaches (Breiman, 2001; Breiman *et al*., 1984; Zararsiz *et al*., 2017). Similar to the performance of using gene biomarker classification (Perez‐Diez *et al*., 2007; van ‘t Veer *et al*., 2002; Wang *et al*., 2005), the reproducibility of the miRNA biomarkers has been challenged (Dupuy and Simon, 2007; Ein‐Dor *et al*., 2006). The prediction performance of gene and miRNA biomarkers often descended drastically in other independent datasets when one dataset was used as the training dataset for the same disease phenotype. These problems may be caused by cellular heterogeneity within tissues, the racial differentiation of the patients, the measurement error in microarray platforms, and the sample shortages.

The core task of the classification method is how to obtain the best classification feature. Previous studies demonstrated that pathways could be used as a crucial feature in identification and classification of disease‐related biomarkers (Khatri *et al*., 2012). For example, pathway enrichment analysis is widely used to identify core regulatory mechanism of biological processes such as tumorigenesis (Shen *et al*., 2017). Pathways can also be used as diagnosis and prognosis biomarkers (Fey *et al*., 2015). Importantly, topologically supported pathway analysis attracts more attention because the interactions between genes can more accurately elucidate the biological mechanism. Therefore, pathway topological analysis can also be used to sort specific pathways of disease subtypes (Ren *et al*., 2018). In order to mine candidate features for classification, researchers inferred the pathway activity with those member genes, which were in the pathway and function‐related genes. Most of these methods integrated member genes together and calculated a score (pathway activity) of those member genes. For example, Guo *et al*. (2005) inferred the pathway activity by computing the mean and median of the expression values of the member genes. Bild *et al*. (2006) inferred the pathway activity by using the first principal component of the expression values of the member genes. To infer the pathway activity, Lee *et al*. (2008) used condition‐responsive genes (CORGs), which combined the expression of the most discriminative power member genes for the disease phenotype. Liu *et al*. (2013) proposed a directed random walk‐based (DRW) method to evaluate the topological importance of each gene and inferred the pathway activity. In addition, there are some probability‐based approaches to estimate the pathway activity (Efroni *et al*., 2007; Su *et al*., 2009). Moreover, a wide variety of high‐throughput omics data were integrated together to detect disease‐specific pathways (Feng *et al*., 2016; Li *et al*., 2017; Shi *et al*., 2016; Vrahatis *et al*., 2016a). For example, Li *et al*. (2017) performed integrative pathway analysis of gene and metabolite to reveal metabolism abnormal regions in the ESCC. Shi *et al*. (2016) presented a new method, which identified dysfunctional pathway by integrating lncRNA–mRNA expression profile and pathway topologies. These methods successfully incorporated different high‐throughput omics data into the disease classification procedures and achieved better classification performance.

The joint impact from genes and miRNAs on disease phenotypes is very important since miRNAs can disrupt biological pathways and cause diseases by targeting genes. Therefore, many studies analyzed miRNA‐mediated pathways by integrating genes and miRNAs (Kretschmann *et al*., 2015; Li *et al*., 2014a). Furthermore, disease phenotypes are found to be highly associated with the key local subpathways, rather than by entire pathways (Li *et al*., 2009, 2013). Focusing on subpathways has been proved to be more effective in identification of disease‐relevant biomarkers (Alaimo *et al*., 2017; Calura *et al*., 2014; Vrahatis *et al*., 2016b; Wang *et al*., 2014; Zhang *et al*., 2017). Therefore, integrating genes and miRNAs at subpathway level might help identification and classification of disease‐relevant biomarkers.

**Figure 1:**
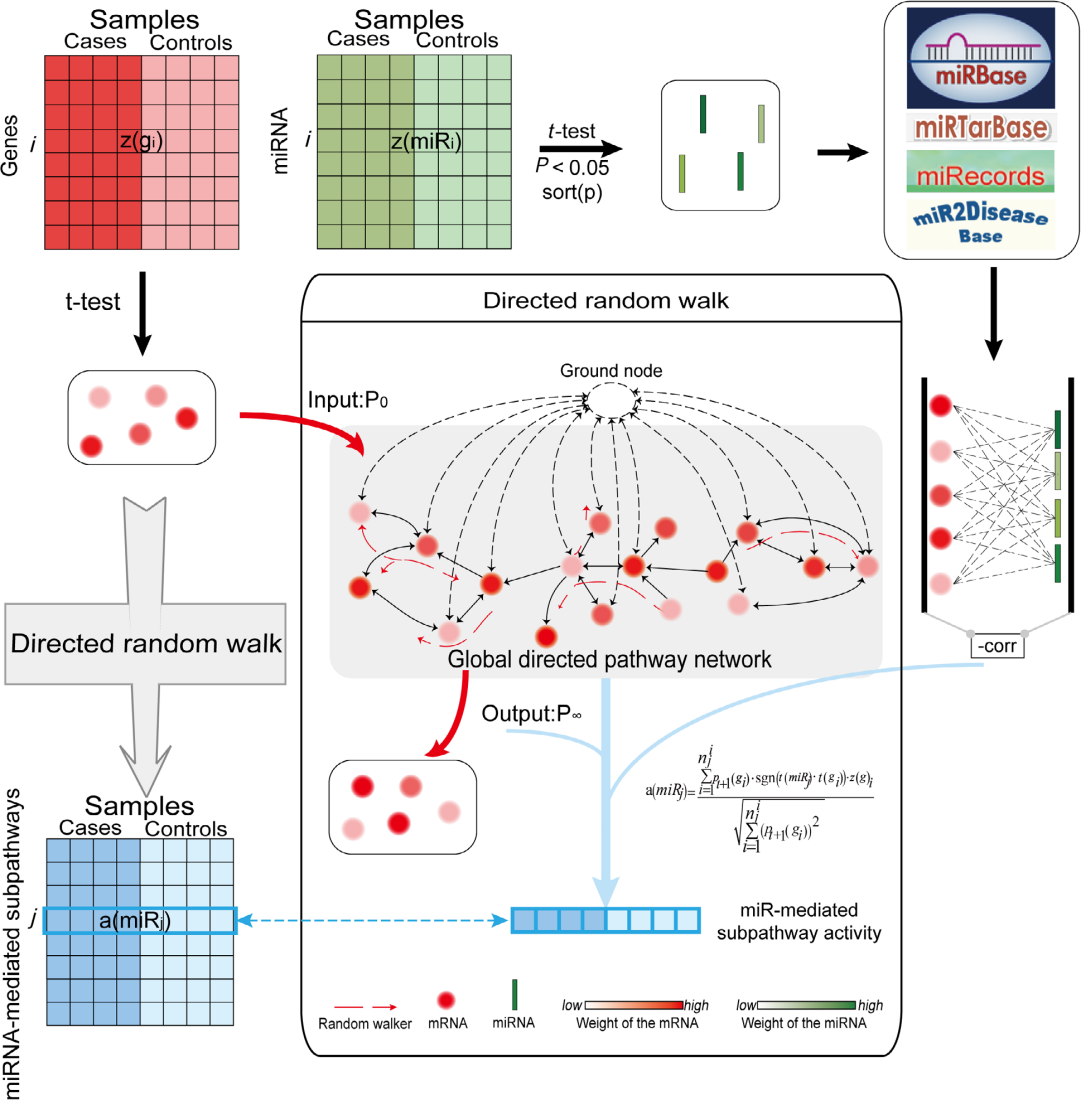
Details of the miDRW‐based miRNA‐mediated subpathway activity inference method. The miRNA‐mediated subpathways are obtained from the gene profiles based on the miDRW method. The *z*(*g*_*i*_) is a row vector of gene *g*_*i*_ expression value across all samples. The *a*(*miR*_*j*_) (i.e., miRNA‐mediated subpathway activity) is also a row vector which is the row *j* of the miRNA (namely *miR*_*j*_) expression value across all samples. The middle panel is the overview illustration of miDRW‐based miRNA‐mediated subpathway activity inference. The GDPN is constructed on 150 metabolic and 150 nonmetabolic pathways, which include 4113 gene nodes and 40 875 directed edges. The dotted line circle is a virtual node which ensures gene weights flow through the GDPN. *P*_0_ is the initial weight of the genes, and *P*_*∞*_ is the output weight vector. For the *miR*_*j*_, we reversed the edge direction when merged the pathways into the GDPN. The miRNA‐mediated subpathway activity *a*(*miR*_*j*_) only integrated expression value vector of the significantly differentially expressed target genes of *miR*_*j*_ into *P*_*∞*_.

Here, we developed a new method that computes miRNA‐mediated subpathway activity by a directed random walk (miDRW). Briefly, miDRW incorporates various information, such as the differentially expressed level of miRNAs and genes, the topological importance of genes in the global directed pathway network (GDPN), clinical information of samples, and target relationships between miRNAs and genes (Fig. 1). The purpose of our method is to topologically infer active miRNA‐mediated subpathways toward precise cancer classification. First, we collected the datasets from TCGA (The Cancer Genome Atlas) and GEO (Gene Expression Omnibus) databases and obtained the target relationships between miRNAs and genes from tarbase V6.0 (Vergoulis *et al*., 2012), miRecords (Xiao *et al*., 2009), miRTarBase (Hsu *et al*., 2011), and miR2Disease (Jiang *et al*., 2009) databases. Second, the GDPN was constructed with genes as nodes. Third, we inferred the expression profiles of miRNA‐mediated subpathway activity and extracted the active miRNA‐mediated subpathways as candidate features with the greedy algorithm. Finally, we compared the performance of the classifier built by miDRW with other existing pathway‐based approaches based on eleven *within‐datasets* and one *cross‐dataset*. The results showed the average values of AUCs are 0.95 and 0.94 on eleven *within‐datasets* and one *cross‐dataset*, respectively. This indicated that the miDRW‐based method could capture active miRNA‐mediated subpathways, which are more reliable clinical biomarkers to classify phenotype.

## 2 Materials and methods

### 2.1 Datasets across cancers

Eleven *within‐datasets* of gene and miRNA expression profiles were obtained from the UCSC Cancer Browser (https://genome-cancer.ucsc.edu), which provided an open‐access portal to download data from TCGA. These normalized TCGA level 3 Illumina HiSeq gene and miRNA expression profiles covered organs of head and neck, breast, liver, lung, kidney, prostate, stomach, thyroid, and uterine corpus (Table S1, Data source). To ensure detection reliability and reduce noise, two filters were applied to eleven sample‐matched datasets of genes and miRNAs. First, miRNAs and genes whose 20% expression value equaled to 0 were eliminated. Second, we selected those datasets that had more than twenty differentially expressed miRNAs (Student's *t*‐test method, *P *< 0.05) and sample‐matched datasets for further analysis.

One *cross‐dataset* of gene and miRNA expression profiles were obtained from the GEO (http://www.ncbi.nlm.nih.gov/geo/). Gene and miRNA expression profiles of the prostate cancer, which were detected on GPL5188 and GPL8227 platforms (GSE21034 and GSE21036), contained sample‐matched information on 28 normal controls and 111 cancer samples (Table S1, Data source). We considered the average expression of genes or miRNAs, which were repeated in the expression profiles. We implemented the same filtering process that was used in *within‐dataset* experiments. Finally, we identified 2427 differentially expressed genes and 68 differentially expressed miRNAs shared by the TCGA and GEO datasets.

### 2.2 miRNAs associated with cancers

More and more studies have confirmed that gene expressions can be regulated not only by neighbor genes, but also by miRNAs. In this study, the target relationships between miRNAs and genes were derived from TarBase (Vergoulis *et al*., 2012), miRecords (Xiao *et al*., 2009), miRTarBase (Hsu *et al*., 2011), and miR2Disease (Jiang *et al*.,^2009^) databases. After we removed redundancy, a total of 755 226 nonrepeated human‐specific interactions among 1137 miRNAs and 20 263 genes were obtained as follows: 598 pairs from miR2Disease, 1749 pairs from miRecords, 26 388 pairs from tarbase (V6.0), and 750 381 pairs from miRTarBase. We integrated predicted and experimentally verified miRNA–gene relationships in our study.

### 2.3 Constructing the global directed pathway network (GDPN)

There were many methods to convert the pathways into graphs (Judeh *et al*., 2013; Sales *et al*., 2012; Vrahatis *et al*., 2016b). These methods used the interaction or regulation relationships between genes to convert pathways into graphs. We used the ‘subpathwayminer v3.0’ (Li *et al*., 2009) software package (http://www.idg.pl/mirrors/CRAN/web/packages/SubpathwayMiner/ or https://github.com/chunquanlipathway) to convert each KEGG pathway into a directed graph. First, those KGML files were converted (KEGG Markup Language, http://www.genome.jp/kegg/xml/) into list variables. In both cases, two genes were connected by an edge: (a) if a common compound existed in their corresponding reaction in a metabolic pathway; and (b) if two genes had a relationship such as interaction, binding, or modification in a nonmetabolic pathway. Thus, with the ‘subpathwayminer v3.0’ software package, we obtained the reconstructed pathway graphs, which retained the topological structure of each pathway. Finally, the total 300 graphs were merged into a global directed pathway graph, which contained 150 metabolic and 150 nonmetabolic pathways. Moreover, we added a virtual node to the global directed pathway graph. The virtual node pointed to each node of the directed pathway graph, and all nodes of the directed pathway graph also pointed to the virtual node (Liu *et al*., 2013). The global directed pathway graph was closed by the virtual node, so we called it the closed global directed pathway network (GDPN), which covered 4113 nodes and 40 875 directed edges. Each node represents a gene, and each directed edge represents interaction or regulation relationships between genes in the GDPN. The direction of the edge is derived from the type of interaction between two genes, which is available from KEGG. For example, if gene A inhibits or activates gene B, the direction of edge pointed to B. The random walk (Lovasz, 1996) on the GDPN is similar to the PageRank algorithm, which is used by the Google search engine (Brin and Page, 1998). The basic idea of the PageRank algorithm is that a web page is important if more other pages point to it. However, a gene is important if it influences more other genes (Draghici *et al*., 2007). Thus, we reversed the directions of all edges in the GDPN (Fig. 1). The GDPN is a biological network, which should be significantly different from random networks (Maslov and Sneppen, 2002). The result showed that the distributions of node degree approximately followed power law distributions with an *R* ^2^ = 0.715, 0.78, and 0.707 for the in‐degree, out‐degree, and total degree, respectively. Only a limited fraction of gene nodes has higher degrees in the GDPN, which is one of the most important basics of random walk algorithm (Watts and Strogatz, 1998).

### 2.4 Calculating node topology score in the GDPN

A node topology score was defined to reflect the topological importance of each gene in the GDPN. We used the directed random walk algorithm to calculate the node topology score with the gene expression value in the GDPN (Eqn 1). The directed random walk algorithm simulated an iterative walker from its source node to a randomly selected immediate neighbor, or at the current node at each time step in the graph. This algorithm could be used to capture global topological relationships within the GDPN and to compute the proximity between the nodes. The formula with restart is defined as follows:

(1) $$p_{t\;+\;1}=\left(1-r\right)M^{T}\;p_{t}+rp_{0}$$

where *M* is the row‐normalized adjacency matrix of the GDPN. The exact approach is to divide the sum of all elements in the row by each element of the row; *p*_*t*_ is a vector in which the *i*th element holds the probability of being at node (genes) *i* at time step *t*. The parameter *r* is the restart probability, which has been demonstrated to have only a slight effect on the results of the directed random walk algorithm (Kohler *et al*., 2008). In this study, the restart probability *r* was set as 0.7.

To start this algorithm, |*t*−score | (the absolute *t*‐test score was called *t*‐score in the following paper) was assigned to each node (except for the virtual node, whose initial probability was 0), and the initial probability vector *p*_0_ was constructed and normalized to a unit vector. After several steps, the probability *p*_*t*_ will converge to a unique steady state *p*_∞_. This steady state was obtained by the iteration until the $∥p_{t+1}-p_{t}∥\leq 10^{-10}$ . The node topology score can be measured by the steady state *p*_∞_, which provided a measure of the topological importance of the genes in the GDPN and was used as the weight vector of genes at the step of miRNA‐mediated subpathway activity inference. We used *t*‐scores as the initial probabilities, and the magnitude of the *t*‐score also contributed to weight adjustments. Thus, the genes will obtain higher topological weights, which are both topologically important and significantly differentially expressed.

### 2.5 miRNA‐mediated subpathway activity inferences

We identified a list of differentially expressed miRNAs (*t*‐test, *P*‐value < 0.05) whose expression level had inverse correlations with significantly differentially expressed target genes (*t*‐test, *P*‐value < 0.05) in the GDPN. Thus, the target genes of each miRNA could be integrated into a special value, which was called miRNA‐mediated subpathway activity. Consider a miRNA‐mediated subpathway miR_*j*_ that targets *n*_*j*_ differential genes $\left\{g_{1},g_{2},\ldots ,g_{n_{j}}\right\}$ . The miRNA‐mediated subpathway activity *a*(*miR*_*j*_) of the *j*th miRNA is calculated as follows:

(2) $$a\left(miR_{j}\right)=\frac{\sum_{i=1}^{n_{j}}p_{\infty }\left(g_{i}\right)\cdot \left[\text{sgn}\left(t\left(miR_{j}\right)\cdot t\left(g_{i}\right)\right)\right]\cdot z\left(g_{i}\right)}{\sqrt{\sum_{i=1}^{\mathrm{nj}}{\left(p_{\infty }\left(g_{i}\right)\right)}^{2}}}$$

*p*_∞_ (*g*_*i*_) is the final weight of *g*_*i*_; *t*() is the *t*‐score for *miR*_*j*_ or *g*_*i*_ from a two‐tailed *t*‐test with expression values between normal and disease samples; and *z* (*g*_*i*_) is the normalized expression value vector of genes across the samples; contrary to common sign function, the sgn() returns +1 for negative numbers and −1 for positive numbers. However, we only consider the negative regulation between miRNAs and genes. For example, for upregulated miRNAs, we only integrated downregulated target genes into the gene set and calculated miRNA‐mediated subpathway activity, and vice versa. Thus, the calculation of *a*(*miR*_*j*_) does not contain the case of same sign (positive regulation). Each miRNA‐mediated subpathway activity (*a*(*miR*_*j*_)) is the integrative score, which includes expression level, difference level, and topological importance of dysregulated target genes in the GDPN. For a specific cancer, the value of *a*(*miR*_*j*_) represents the influence of the *j*th differentially expressed miRNA on the GDPN. The larger activity value is, the greater impact on the GDPN it has. Therefore, the miRNA with larger value is possible to be the more effective biomarker of the cancer.

### 2.6 Classification evaluation

For *within‐dataset* experiments, we randomly selected one‐fifth of samples for test and the rest for training (fivefold cross‐validation). To identify the most effective miRNA‐mediated subpathways for classification, we further divided the training set into three subsets of equal size. Two subsets were used as the *Feature Retrieve subset* to establish the classifier, and the remaining one subset was used as the *Feature Selection subset* for optimizing the classifier and select features (classification biomarkers). We ranked miRNA‐mediated subpathway activities of the *Feature Retrieve subset* in ascending order by calculating the *P*‐values of two‐tailed *t*‐test statistics. The top biomarkers were used as the candidate features. For fairly evaluating the performance of methods, we implemented the methods with the same number of candidate features (miRNA‐mediated subpathways) and recorded the frequency of the miRNA‐mediated subpathways appearing in the results. The features were called high‐frequency (HF) subpathways if the frequency was larger than the median. The top 20 miRNA‐mediated subpathways were used as candidate features to build classifiers (e.g., Logistic regression, Naive Bayes, and J48) in our method. We evaluated the influence of five different thresholds (top 10 to top 50) on the classifier. The results showed there was no substantial improvement for the performance of the classifier when the number of candidate feature exceeded 20 (Fig. S1A,B). We identified candidate features with greedy algorithm. The first classifier was built by the miRNA‐mediated subpathway ranking first, and the remaining 19 miRNA‐mediated subpathways were added to the classifier sequentially, and we recorded the areas under the curve (AUCs). The miRNA‐mediated subpathway was selected as a feature if the AUC increased, or was removed otherwise. After the iterative process, we could obtain one optimized classifier and one feature set. The feature set was used to evaluate the performance of the optimized classifier on the test set. Therefore, three optimized classifiers and three AUCs in the corresponding *Feature Selection subset* generated from each training set. Thus, 15 AUCs were generated from five test sets in turn. For unbiasedly evaluating the performance of the classifier and estimating the fluctuation of the AUCs, we repeated the above process for 10 times. The averaged AUC across 150 classifiers was reported as the overall performance of the classification method.

For *cross‐dataset* experiment, one dataset was used as the training set, and the other independent dataset was used as the test set. The training set was randomly divided into five subsets with equal size. One subset was used as the *Feature Selection subset* to optimize classifier and select features, whereas the remaining subsets were used as the *Feature Retrieve subset* to establish the classifier. For unbiased evaluation, we built the classifier with the differentially expressed miRNAs and target relationships of the training set, and repeated the above process 10 times by using each subset as the *Feature Selection subset* in turn and evaluated the optimized classifier on the test set. The averaged AUC across 50 classifiers was reported as the overall performance.

### 2.7 Reproducibility power

We consider the training–validation datasets to be reproducible if their miRNA‐mediated subpathway activities provide similar discriminative power (Yang *et al*., 2012). Then, we define reproducibility power by

(3) $$C_{\mathrm{score}}\left(N\right)=\frac{1}{N}\sum_{i=1}^{N}t\left(miR_{t}^{i}\right)t\left(miR_{v}^{i}\right)$$

*t*(*miR*) is the *t*‐score (*t*‐test, absolute *t*‐value) of miRNA‐mediated subpathway activities between cancer and normal samples. $miR_{t}^{i}$ and $miR_{v}^{i}$ are the *i*th miRNA‐mediated subpathway activities of the training dataset and the validation dataset, which are ranked by *t*‐scores in ascending order. *N* is the number of selected miRNA‐mediated subpathways. The reproducibility power reflects the discriminative power and the robustness of the miRNA‐mediated subpathway activity. For *within‐dataset* experiments, we randomly divided the samples into five equal‐sized subsets. Each subset was used in turn as the test set to evaluate the reproducibility, whereas the remaining subsets were used as the training set. For unbiased evaluation, we repeated the above procedure for 100 times. It was reported as the overall reproducibility with the averaged *C*_*score*_ over 500 experiments.

For *cross‐dataset* experiments, one dataset was used as the training dataset, and the other independent dataset was used as the test set with the same experimental procedure.

## 3 Results and Discussion

### 3.1 Inferring subpathway activity and evaluating classification performance

The general idea of the miDRW is depicted in Fig. 1. miDRW calculates the topology score of genes according to their topological importance in the GDPN. Then, we computed the activity of each miRNA‐mediated subpathway by incorporating the expression of genes, the differential level of miRNAs and genes, the topology score of genes, and the target relationships between miRNAs and genes. The more topologically important the genes are, the larger topology score they can obtain, and the larger activities they contribute to the miRNA‐mediated subpathway. Finally, the miDRW method was used to convert expression profiles of genes into miRNA‐mediated subpathway activity profiles (see Section 2).

**Figure 2:**
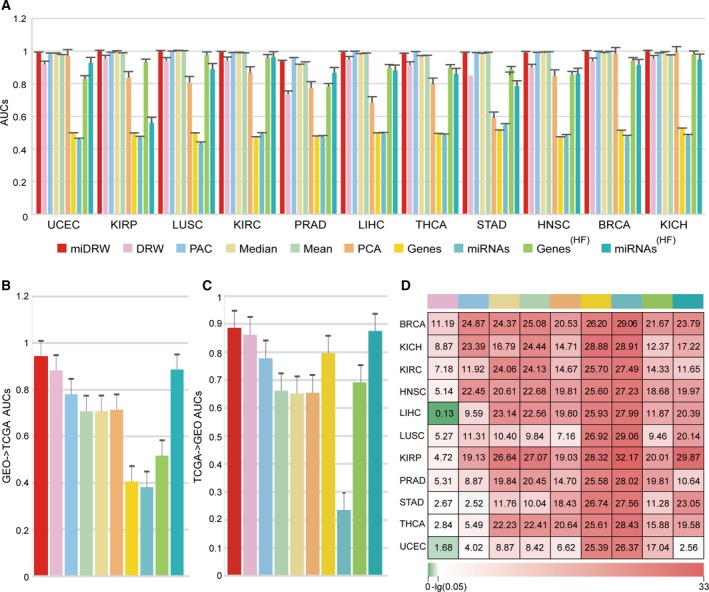
Classification performances of Logistic regression. (A) The height of the bar represents the AUCs which are generated by Logistic regression on *within‐datasets*. (B) The height of the bar represents the AUCs which are generated by Logistic regression on the ‘GEO‐>TCGA’ *cross‐dataset*. (C) The height of the bar represents the AUCs which are generated by Logistic regression on the ‘TCGA‐>GEO’ *cross‐dataset*. (D) A global view of the statistical significances for 11 *within‐datasets*. Rows represent cancers, and columns represent methods. Values represent the −log10 (p) of the Wilcoxon signed‐rank test between the AUCs of miDRW and the AUCs of other methods. Error bars represent standard deviation in (A), (B), and (C).

For comparison with other miRNA‐mediated subpathway‐based methods, we manually retrieved a lot of literatures, whereas there were no similar methods. So we implemented five famous pathway‐based classification methods that contained the Mean and Median methods (Guo *et al*., 2005), the PCA method (Bild *et al*., 2006), the PAC method (Lee *et al*., 2008), and the previous DRW method (Liu *et al*., 2013). To test the discriminative power of miRNA‐mediated subpathway activities between normal and cancer samples, we performed *within‐dataset* experiments similar to those used in Lee *et al*. (2008) to evaluate the classification performance of eleven TCGA datasets with 10 times fivefold cross‐validation (see Section 2). We used the top 20 pathway activities (our methods selected the top 20 miRNA‐mediated subpathway activities, *p*‐values in ascending order) as the candidate features to build the classifier with Logistic regression for the Mean and Median methods, PAC method, PCA method, DRW method, and miDRW method. Additionally, we investigated the performance of the traditional gene‐based and miRNA‐based classifiers that used genes and miRNAs as markers. For the gene‐based or miRNA‐based methods, we used not only the top 20 discriminative genes (Genes method) and miRNAs (miRNAs method), but also all genes (Genes^(HF)^ method) and miRNAs (miRNAs^(HF)^ method) incorporated in the top 20 miRNA‐mediated subpathway activities to evaluate the performance of classification (Fig. 2A, Fig. S2A).

Figure 2 depicts a summary of AUCs of the *within‐datasets* and *cross‐dataset* experiments. The miDRW method calculated the average AUCs (accuracies) (Fig. 2A, Fig. S2A) as 0.9885 (0.9741) based on all *within‐datasets*. The average AUC of miDRW method had the minimum standard deviation of 0.009 among all *within‐datasets* (Table S1, Logistic *within‐datasets*). The AUCs of the miDRW method on the eleven within‐datasets were significantly greater than those of the DRW (except LIHC), PAC, Mean, Median, PCA, Genes, miRNAs, Genes^(HF)^, and miRNAs^(HF)^ methods (Wilcoxon signed‐rank test; Fig. 2D). Similarly, the accuracies of the miDRW were also statistically significant, compared with other methods (Wilcoxon signed‐rank test; Fig. S2D). This indicated that the miDRW method outperformed other methods not only in AUC but also in accuracy. These results showed that the miRNA‐mediated subpathways of miDRW‐based method were quite capable of discriminating different phenotypes (normal vs. cancer). It is noted that the miDRW method obtained the highest AUCs from seven *within‐datasets* and the second AUCs from the remaining *within‐datasets*. Meanwhile, the average value of AUCs outperformed than other classification methods on *within‐datasets*. The overall trend of accuracies is similar to AUCs (Fig. S2A). This indicated that miRNA‐mediated subpathways inferred by the miDRW method were more discriminative, and the performance of the classifier was more stable (Table S1, Logistic *within‐datasets*). This result also revealed that the topologically inferred active miRNA‐mediated subpathway was an effective integration strategy for classification problems.

Furthermore, we evaluated the generalization ability of the classifier by carrying out the *cross‐dataset* experiment on the prostate adenocarcinoma samples. First, the GEO prostate adenocarcinoma dataset was used as the training set and TCGA prostate cancer dataset was used as the test set (see Section 2); then, their roles were swapped. The average AUC and accuracy of ‘CEO‐>TCGA’ and ‘TCGA‐>GEO’ are 0.9157 and 0.8747 (Table S1, Logistic *cross‐dataset*), respectively. The AUCs (accuracies) of the miDRW method on the cross*‐dataset* were significantly greater than those of the DRW, PAC, Mean, Median, PCA, Genes, miRNAs, Genes^(HF)^, and miRNAs^(HF)^ methods (Wilcoxon signed‐rank test of TCGA‐>GEO: *p*‐value=1.41e‐9 (8.86e‐8), 5.18e‐9 (6.99e‐2), 1.41e‐9 (8.83e‐11), 1.41e‐9 (9.36e‐9), 1.41e‐9 (7.75e‐4), 1.41e‐9 (2.31e‐9), 9.99e‐5 (1.26e‐2), 1.41e‐9 (2.31e‐9), 9.99e‐5 (1.27e‐2); Wilcoxon signed‐rank test of GEO‐>TCGA: *p*‐value=4.74e‐4 (3.57e‐2), 3.85e‐9 (5.65e‐4), 1.40e‐9 (1.94e‐7), 1.40e‐9 (2.04e‐7), 7.30e‐9 (6.90e‐9), 1.40e‐9 (2.00e‐8), 7.79e‐5 (4.98e‐2), 1.40e‐9 (2.00e‐8), 7.79e‐5 (4.98e‐2); Fig. 2B,C, Fig. S2B,C). Specifically, two prostate adenocarcinoma datasets were detected on different measurement platforms and cohorts of patients. This indicated that the miDRW‐based method not only considered topological importance and differential expression of genes in the GDPN, but also integrated the target relationships between miRNAs and genes to construct the classifier. Therefore, the miDRW method, which can predict more practical markers in clinical applications, has stronger generalization ability and discriminative power.

Finally, we repeated the *within‐dataset* and the *cross‐dataset* experiments to prove that the good performance of the miDRW method was not dependent on specific classification algorithms by using the Naive Bayes model (John and Langley, 1995) and J48 (Chen *et al*., 2014; Jagga *et al*., 2014). It is not surprising that we obtained similar results to the Logistic regression classification algorithm (Table S1, Naive Bayes *within‐datasets*, J48 *within‐datasets*).

### 3.2 The miDRW method applied to KICH within‐dataset

**Figure 3:**
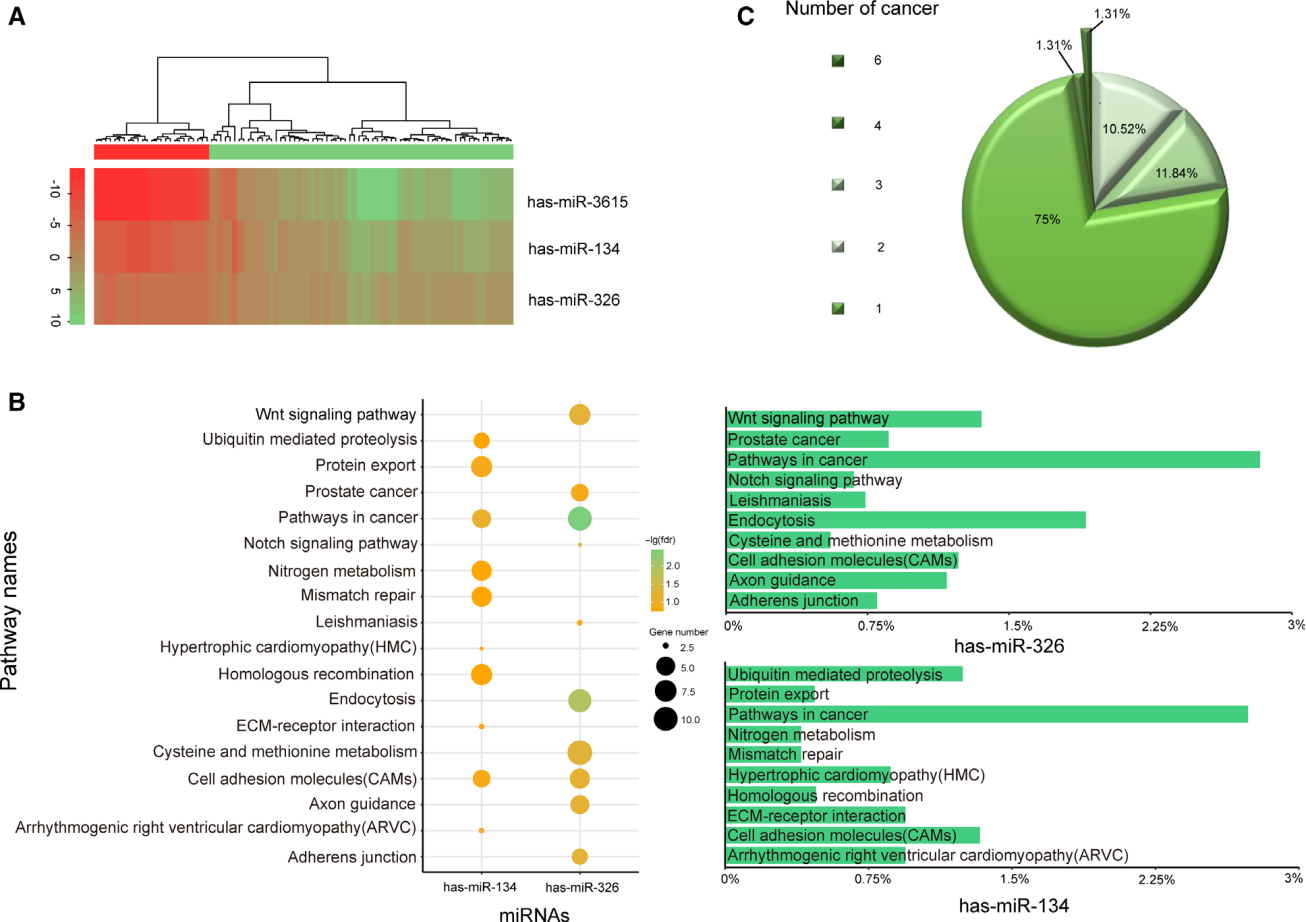
The selected active miRNA‐mediated subpathways are associated with important pathways. (A) The hierarchical cluster analysis based on active miRNA‐mediated subpathways of KICH before the median frequency. The row and column represent miRNA‐mediated subpathway and samples (the red and green bars represent normal and cancer samples), respectively. (B) The summary bubble‐bar plot shows the functional enrichment results of the active miRNA‐mediated subpathways of KICH. The bars on the right show the percentage of significantly differentially expressed genes annotated to the KEGG pathways. The bubble size indicates the number of genes in each KEGG pathway, and different colors correspond to different FDRs. The darker color indicates the smaller FDR. (C) The pie chart shows the proportion of active miRNA‐mediated subpathways present in different cancers. The majority of the active miRNA‐mediated subpathways are cancer‐specific.

**Figure 4:**
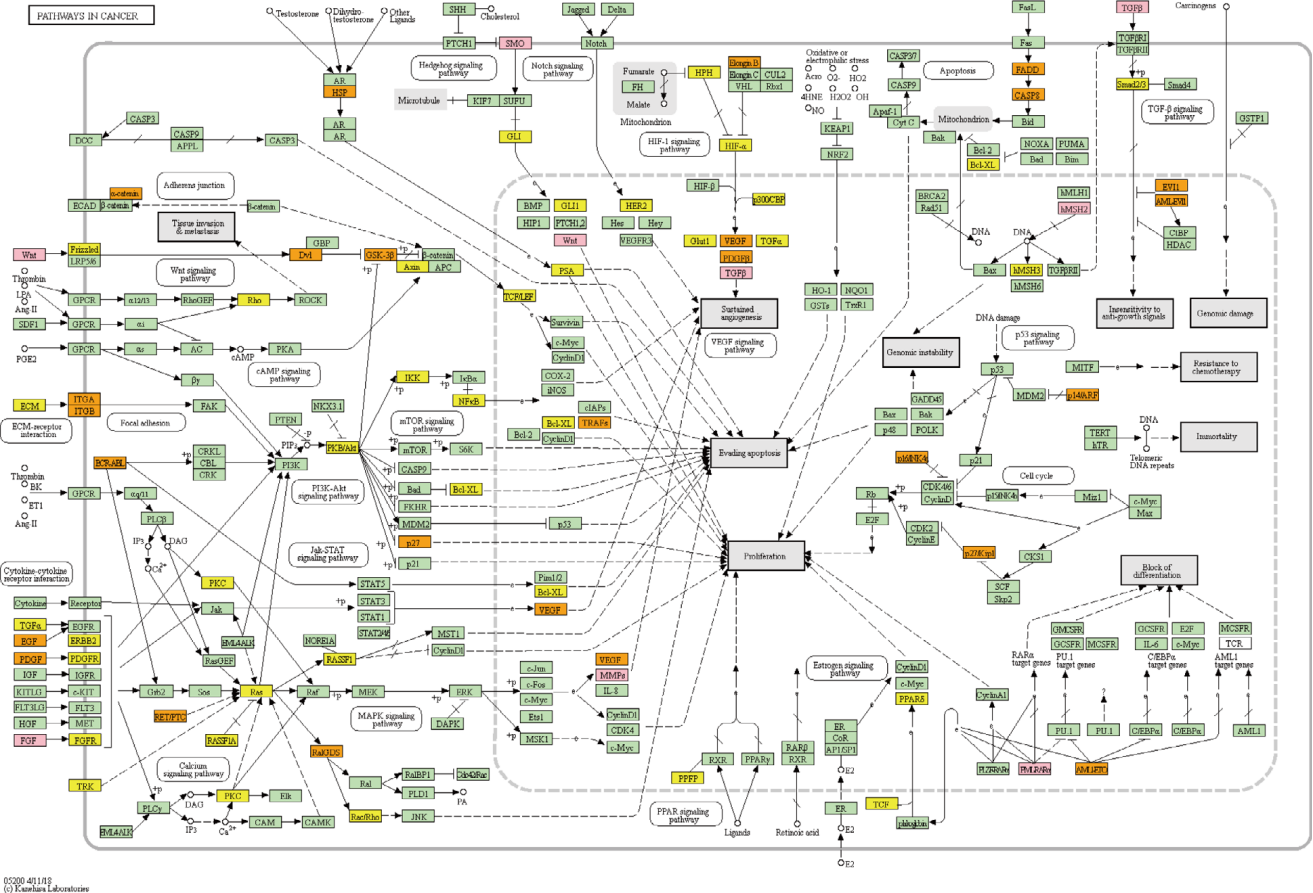
A snapshot of the pathways in cancer (hsa05200). The orange (yellow) color nodes represent the differentially expressed target genes of hsa‐miR‐134 (hsa‐miR‐326). The pink color nodes represent the common differentially expressed target genes of hsa‐miR‐134 and hsa‐miR‐326.

We applied the miDRW method to the KICH *within‐datasets* (Table S1, Logistic *within‐datasets*) and obtained 18 miRNA‐mediated subpathways. The three high‐frequency miRNA‐mediated subpathways were able to accurately classify samples by complete hierarchical clustering (Fig. 3A). The high‐frequency miRNA‐mediated subpathways of the remaining 9 *within‐datasets* also showed good discriminative ability, even for highly heterogeneous BRCA (Fig. S3A‐I). However, the HNSC showed an unsatisfactory clustering effect because the *within‐datasets* included multiple subtypes (Fig. S3J). Moreover, the differentially expressed target genes of hsa‐miR‐134 and hsa‐miR‐326 were annotated to cancer‐related pathways except hsa‐miR‐3615 in the KICH, which contained only few differentially expressed target genes (member genes, *P*‐value < 0.01 and FDR < 0.01; Fig. 3B,C). For the annotated pathways by target genes of hsa‐miR‐134 and hsa‐miR‐326, we ranked them with the *p*‐value in ascending order, respectively, and selected the top 10 of pathways. Pathways in cancer (hsa05200) and cell adhesion molecules (CAMs, hsa04514) were common pathways of annotated results (Fig. 4, Fig. S4). In renal cell carcinoma, hypoxia‐inducible factor (HIF‐) transcription factor accumulates, resulting in the overexpression of proteins that are normally inducible with hypoxia, such as transforming growth factor (TGF‐ and TGF‐, respectively) and vascular endothelial growth factor (VEGF), and platelet‐derived growth factor β chain (PDGF‐β). The overexpressed VEGF, PDGF‐β, and TGF‐ act on neighboring vascular cells to promote tumor angiogenesis (Cohen and McGovern, 2005). TGF was the common target gene of hsa‐miR‐134 and hsa‐miR‐326 in the KICH, which promoted cancer‐cell proliferation and survival. For the cell adhesion molecules pathway, an improved understanding of immunobiology uncovered the importance of immune checkpoints in facilitating tumor escape, leading to the development of multiple novel therapeutics targeting PD‐L1 (programmed death‐ligand 1, CD274) immune checkpoints (Patel and Kurzrock, 2015). In addition to the genes annotated to the above two pathways, there are also the driver genes annotated to the renal cancer‐related pathways, such as AKT2 (Guo *et al*., 2015) and TCEB1 (Hakimi *et al*., 2015). Similarly, for each high‐frequency miRNA‐mediated subpathway, we obtained the member genes of the subpathway and annotated these genes to the KEGG pathways. The results showed that the majority of these genes appeared in cancer‐related pathways (Table S2, Fig. S5A–H). Moreover, the pathway‐related miRNAs were annotated to the corresponding cancers in the HMDD (Human microRNA Disease Database v3.0; Huang *et al*., 2019; Fig. S6, Table S3). As shown in Fig. 3C, only a small fraction of miRNAs was shared by different cancers, and most of the miRNAs were cancer‐specific.

The above results indicated that these high‐frequency subpathways were active in cancers and discriminative for classification. One reason might be that the active miRNA‐mediated subpathway was integrated by cancer‐relevant genes. This integrative strategy considered differential expressions, differential levels, topological information, and target relationships. Therefore, these miRNA‐mediated subpathways could be used as classification biomarkers.

### 3.3 miRNA‐mediated subpathways show high reproducibility

**Figure 5:**
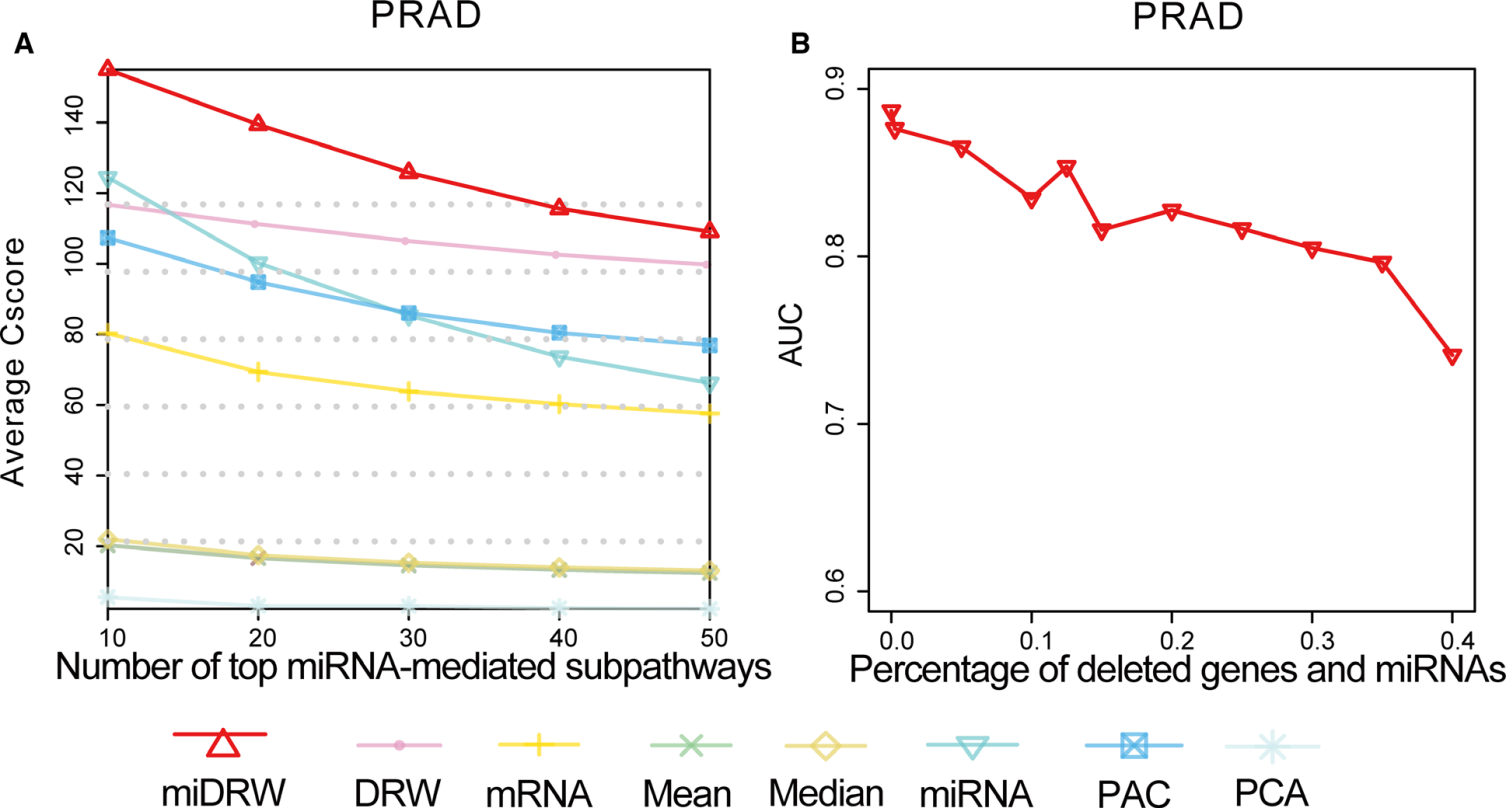
The influence of topological structure and reproducibility power of the miDRW method for *within‐dataset* experiments. (A) The line indicates the reproducibility power of the miDRW method for *within‐dataset* experiments. The *x*‐axis represents the number of top miRNA‐mediated subpathways, and the *y*‐axis shows the reproducibility power *C*_score_ of the top *k* miRNA‐mediated subpathways, *k *= 10, 20, 30, and 40. (B) The line shows the influence of topological structure and target relationships on the miDRW method. The *x*‐axis represents the percentage of deleted target genes and miRNAs, and the *y*‐axis shows the AUCs obtained corresponding to those percentage.

In our previous study, the DRW method inferred the pathway activity by integrating the gene expression profiles and obtained stronger discriminative power and robustness active pathways. In this study, we upgraded the DRW method by considering the miRNA expression profiles and the target relationships between miRNAs and genes. The greater reproducibility power indicates more generalization ability of classifier and stronger robustness of miRNA‐mediated subpathways. We calculated the reproducibility power according to formula (3) and ranked the miRNA‐mediated subpathways in descending order on each *within‐dataset*. We compared the mean reproducibility power of the miDRW‐based method and pathway‐based methods. Moreover, we also evaluated the reproducibility power of the top genes and miRNAs. The genes were chosen from the 4113 genes covered by the GDPN, and the miRNAs were differentially expressed between normal and cancer samples. The reproducibility of miRNA‐mediated subpathway activities exhibited the greatest power to distinguish normal from cancer samples for three datasets (PRAD, STAD, and UCEC). The PAC method obtained the greatest reproducibility power only on the LUSC dataset. The DRW‐based method obtained the highest reproducibility power on the remaining datasets (Fig. 5A, Fig. S7A–J). For the cross‐dataset reproducibility experiments, our method was slightly inferior to the DRW‐based method (Fig. S7K). Moreover, the AUCs and accuracies of the miDRW‐based method were higher than those of the DRW‐based method (Fig. 2B,C, Fig. S2B,C). It implied that the biomarkers were more reproducible by topologically inferring, and the miDRW‐based method outperformed the DRW‐based method.

### 3.4 Robustness of high active miRNA‐mediated subpathways

We built the classifier with frequently selected miRNA‐mediated subpathways, which may be new, robust active markers for cancers. The TCGA and GEO datasets of prostate adenocarcinoma shared 180 miRNAs, 68 of which were significantly different (*P*‐value < 0.05). Then, we repeated the *within‐dataset* experiments based on the differential miRNAs and their differential target genes of TCGA and GEO datasets. We obtained 9 and 27 robust active miRNA‐mediated subpathways, respectively. Finally, 4 miRNA‐mediated subpathways were frequently selected by *within‐dataset* experiments from TCGA and GEO datasets, including hsa‐miR‐96, hsa‐miR‐133b, hsa‐miR‐192, and hsa‐miR‐136 (hypergeometric test, *P*‐value = 0.004). The null hypothesis of the hypergeometric test was no overlap between the miRNA‐mediated subpathways of the TCGA and GEO datasets. If the *P*‐value is < 0.05, we reject the null hypothesis, which means that these miRNA‐mediated subpathways could be used as classification biomarkers to classify samples. It is reported that miR‐96 usually functions as an oncogene during tumorigenesis, which is overexpressed in prostate cancer (Guo *et al*., 2012, 2014; Haflidadottir *et al*., 2013; Xu *et al*., 2013). It has been shown that overexpression of miR‐133b in LNCaP cells boosted cell proliferation and cell‐cycle progression, and miR‐133b might be independent prognostic factors of biochemical recurrence (Li *et al*., 2014a,b). Sun *et al*. (2016) indicated that miR‐192 negatively regulated NOB1 expression and impaired the tumorigenicity of prostate cancer cells.

To test the influence of the topological structure on the classifier, we randomly deleted the 5–40% of the target genes in the GDPN and miRNAs in turn. The results of the *cross‐dataset* experiment in the ‘TCGA‐>GEO’ case demonstrated that the AUC decreased dramatically when the percentage exceeded 35%, confirming the importance of topological network (Fig. 5B). Results indicated that the miDRW‐based method was capable of identifying more robust active and cancer‐related miRNA‐mediated subpathways, though the datasets came from different measurement platforms and patient cohorts. Meanwhile, the miDRW method can resist certain topological damage and has good robustness.

To test the influence of the target relationships between miRNAs and genes on the classifier, we performed the deleting experiment on the *within‐datasets*. We randomly deleted 10%, 20%, 30%, 40%, and 50% of all target relationships, which were disease‐specific target pairs of differentially expressed miRNAs and genes. The AUCs and accuracies of classifiers decreased slowly with the increase in the percentage of deleting the target relationships (Fig. S1C,D). For example, even though we deleted 50% of all target relationships, the AUCs and accuracies of classifiers on within‐datasets were still larger than 0.8. These results implied that the performance of our method was stable. A reasonable explanation is that our method obtained miRNA‐mediated subpathway activities by integrating multi‐omics data with topological information. Moreover, the miDRW method could assign more weights to differentially expressed genes, which were the targets of the miRNA‐mediated subpathway in the GDPN. Therefore, the classifier can robustly classify phenotypes with reproducible miRNA‐mediated subpathway activities.

## 4 Conclusions

In conclusion, our findings showed that high active miRNA‐mediated subpathways improved cancer classification performance and showed high reproducibility between the training set and the test set. Moreover, the miDRW method did not depend on specific classification algorithms. We computed the activity of each miRNA‐mediated subpathway by incorporating the expression of genes, the differential levels of miRNAs and their target genes, topological importance of differential target genes, and clinical information of matched samples. Therefore, the miDRW method can significantly reduce noise from sequencing errors and samples’ heterogeneity by integrating pathway topological information. However, our method depends on the data collection and credibility of target relationships. Thus, with the rapid development of human interaction databases and the sequence technology, we believe that the miDRW method is a promising way to precisely predict the state of disease and provide a better guide for patient treatment.

## Conflict of interest

The authors declare no conflict of interest.

## Author contributions

ZN, JZ, and CL developed the study concept and design and extracted data from database. ZN performed training of algorithm. ZN drafted the manuscript. CF, CS, WL, DS, and XY critically revised the draft manuscript. CL had full access to all the data in the study and takes responsibility for the integrity of the data and the accuracy of the data analysis. All authors reviewed the manuscript.

## Supporting information


**Fig. S1.** The influence of thresholds and target relationships deleting on the classification performance. (A)‐(B) The height of the bar represents the AUCs and accuracies which are generated with different thresholds (top 10‐top 50) of the miDRW method on *within‐datasets*. (C)‐(D) The line indicates the AUCs and accuracies of the miDRW method for *within‐dataset* experiments. *X*‐axis represents the deleted ratio of miRNA and gene pair, and *y*‐axis represents AUCs and accuracies. The error bars represent standard deviation in (A) and (B).

**Fig. S2.** Classification performances of Logistic regression. (A) The height of the bar represents the accuracies which are generated by Logistic regression on *within‐datasets*. (B) The height of the bar represents the accuracies which are generated by Logistic regression on ‘GEO‐>TCGA’ *cross‐dataset*. (C) The height of the bar represents the accuracies which are generated by Logistic regression on ‘TCGA‐>GEO’ *cross‐dat*aset. (D) A global view of the statistically significant for 11 *within‐datasets*. Rows represent cancers, and columns represent methods. Values represent the ‐log10(p) of the Wilcoxon signed‐rank test between the accuracies of miDRW and the accuracies of other methods. The error bars represent standard deviation in (A), (B), and (C).

**Fig. S3.** The hierarchical cluster analysis based on active miRNA‐mediated subpathways of other cancers before the median frequency. The row and column represent miRNA‐mediated subpathway and samples (the red and green bars represent normal and cancer samples), respectively.

**Fig. S4.** A snapshot of the Cell adhesion molecules (CAMs, hsa04514). The orange (yellow) color nodes represent the differentially expressed target genes of hsa‐miR‐134 (hsa‐miR‐326). The pink color nodes represent the common differentially expressed target genes of hsa‐miR‐134 and hsa‐miR‐326.

**Fig. S5.** The summary bubble‐bar plot shows the functional enrichment results of the active miRNA‐mediated subpathways of other cancers. The bars on the right show the percentage of significantly differentially expressed genes annotated to the KEGG pathways. The bubble size indicates the number of genes in each KEGG pathway, and different colors correspond to different FDRs. The darker color indicates the smaller FDR.

**Fig. S6.** A global view of topologically inferring active subpathways and cancers. Each column represents one cancer, and each row represents an active miRNA‐mediated subpathway.

**Fig. S7.** Reproducibility power of the miDRW method for *within‐datasets* and *cross‐dataset* experiments. (A)‐(J) The line indicates the reproducibility power of the miDRW method for *within‐dataset* experiments. The *x*‐axis represents the number of top miRNA‐mediated subpathways, and the *y*‐axis shows the reproducibility power C_*score*_ of the top *k* miRNA‐mediated subpathways, *k*=10, 20, 30, 40. (K) The line indicates the reproducibility power of the miDRW method for PRAD *cross‐dataset* experiments. The *x*‐axis represents the number of top miRNA‐mediated subpathways, and the *y*‐axis shows the reproducibility power C_*score*_ of the top *k* miRNA‐mediated subpathways, *k*=10, 20, 30, 40.

**Table S1.** Data source and validation results. Data source: The datasets used in evaluation of the miDRW method. Logistic *within‐datasets*: Shown are the average AUCs and average accuracies of *within‐dataset* experiments using Logistic classifier. Naive Bayes *within‐datasets*: Shown are the average AUCs and average accuracies of *within‐dataset* experiments using Naive Bayes classifier. J48 *within‐datasets*: Shown are the average AUCs and average accuracies of *within‐dataset* experiments using J48 classifier. Logistic *cross‐dataset*: Shown are the average AUCs and accuracies of *cross‐dataset* experiments using Logistic classifier.

**Table S2.** The differentially expressed target genes of hsa‐miR‐134 and hsa‐miR‐326 were annotated to pathways of Pathways in cancer and Cell adhesion molecule. We collected relationship between the genes, pathways and cancers from NCBI PubMed.

**Table S3.** The results of collecting the cancer‐associated miRNAs from the HMDD.
