# Corrigendum to: Topologically inferring active miRNA‐mediated subpathways toward precise cancer classification by directed random walk

**DOI:** 10.1002/1878-0261.12590

**Published:** 2019-10-31

In the paper by Ning et al. (2019), a funding section should have been included, which is given below:

## Funding

This work was supported by the Fundamental Research Funds for the Provincial Universities; the Scientific Research Fund of Harbin Medical University‐Daqing (Grant No. DQXN201706); the National Natural Science Foundation of China (Grant Nos 81572341 and 61601150); the Natural Science Foundation of Heilongjiang Province (Grant No. JJ2016ZR1232/F2016031); Yu Weihan Outstanding Youth Training Fund of Harbin Medical University; Wu Liande Youth Science Research Fund of Harbin Medical University (Daqing) (Grant No. DQWLD201703); Innovative Scientific Research Fund of Harbin Medical University (Grant No. 2016JCZX03); and the National Science Foundation of Heilongjiang Province (Grant No. YQ2019C013).
