# Supplementary figures and images for: Topologically inferring active miRNA‐mediated subpathways toward precise cancer classification by directed random walk
Source: Mol Oncol. 2019 Aug 27;13(10):2211–26. doi: 10.1002/1878-0261.12563 (PMC6763789; doi:10.1002/1878-0261.12563)

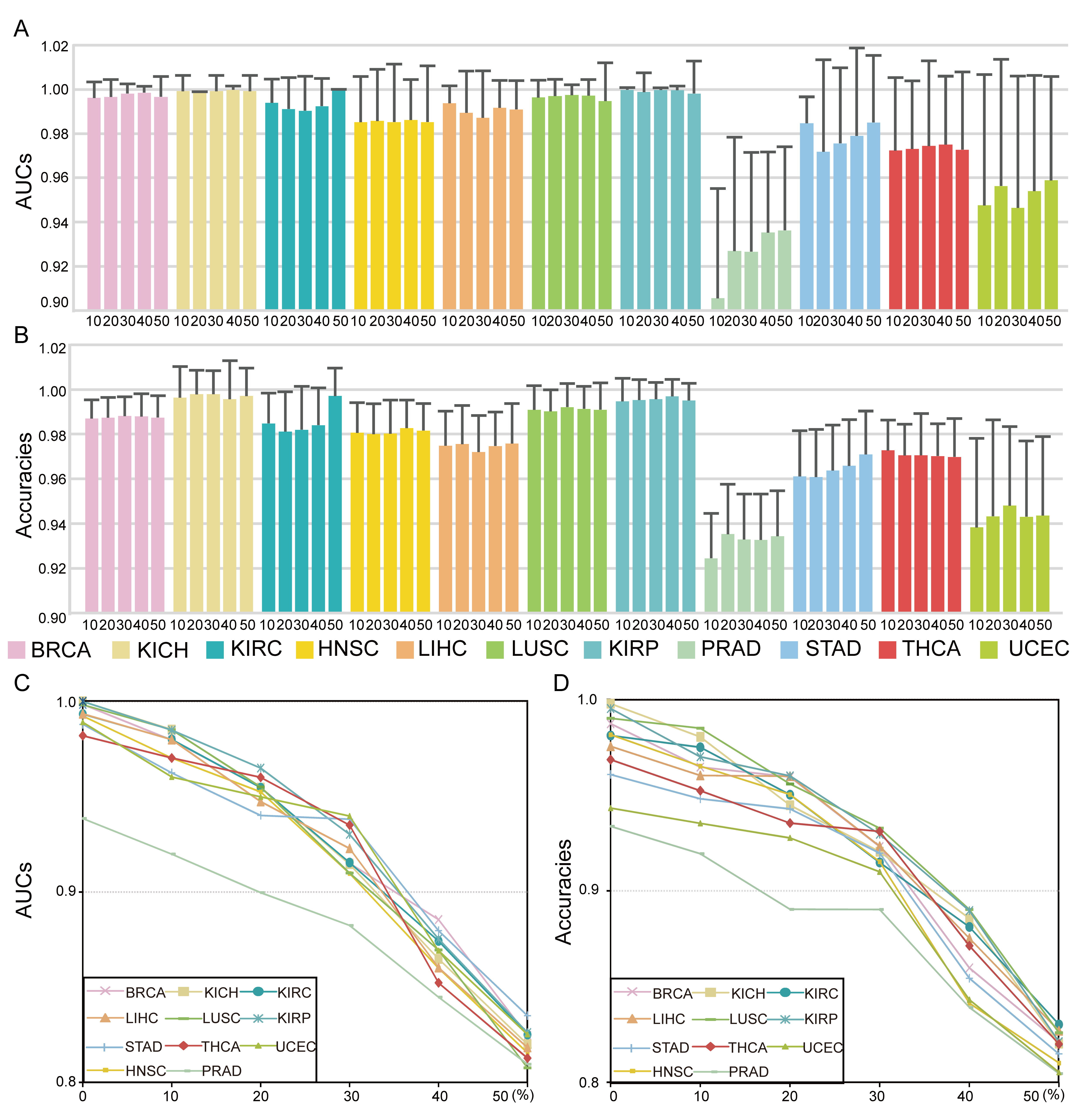

Supplement: Supplementary file 1 — Fig. S1. The influence of thresholds and target relationships deleting on the classification performance. (A)‐(B) The height of the bar represents the AUCs and accuracies which are generated with different thresholds (top 10‐top 50) of the miDRW method on within‐datasets. (C)‐(D) The line indicates the AUCs and accuracies of the miDRW method for within‐dataset experiments. X‐axis represents the deleted ratio of miRNA and gene pair, and y‐axis represents AUCs and accuracies. The error bars represent standard deviation in (A) and (B). [file MOL2-13-2211-s001.tif]

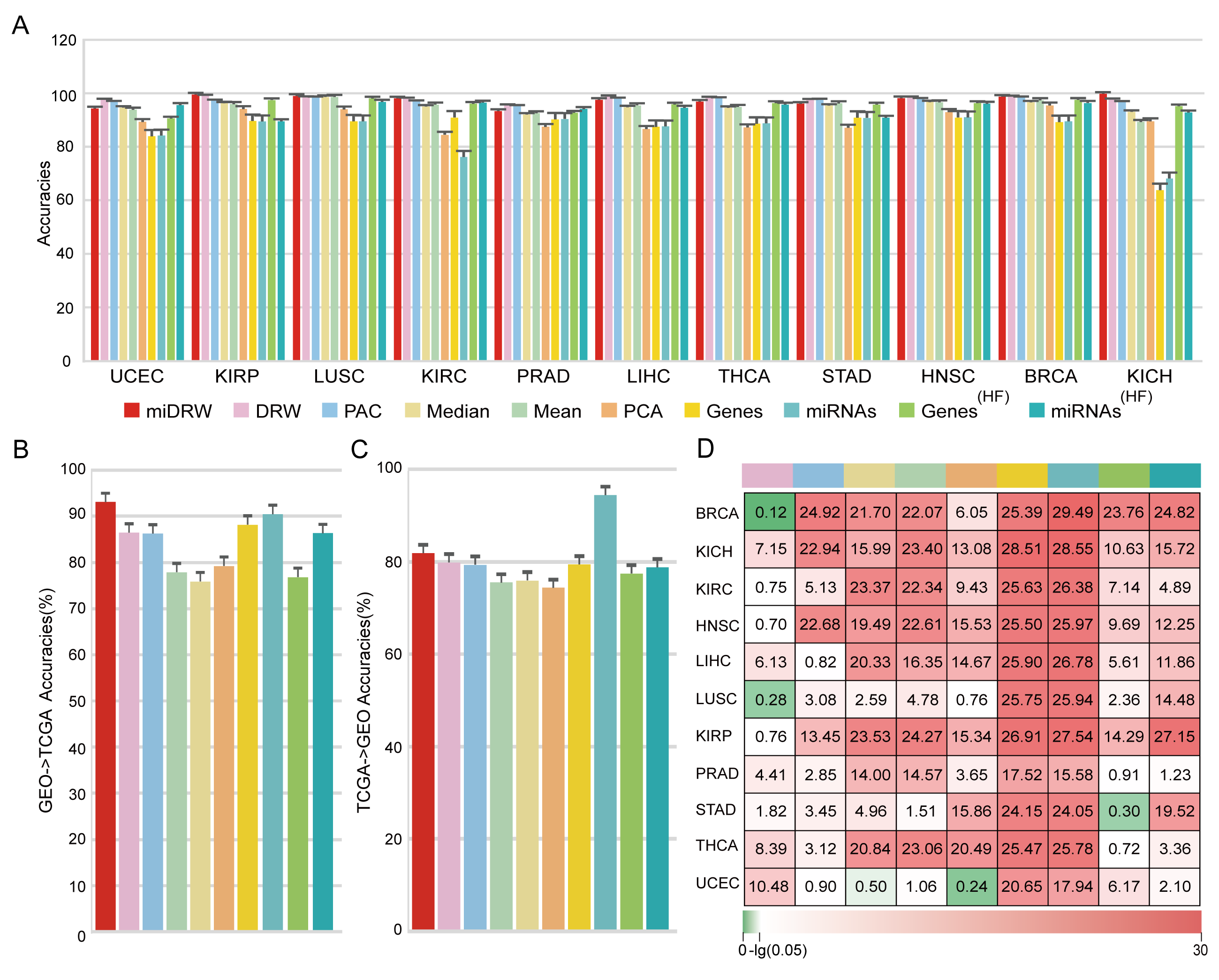

Supplement: Supplementary file 2 — Fig. S2. Classification performances of Logistic regression. (A) The height of the bar represents the accuracies which are generated by Logistic regression on within‐datasets. (B) The height of the bar represents the accuracies which are generated by Logistic regression on ‘GEO‐>TCGA’ cross‐dataset. (C) The height of the bar represents the accuracies which are generated by Logistic regression on ‘TCGA‐>GEO’ cross‐dataset. (D) A global view of the statistically significant for 11 within‐datasets. Rows represent cancers, and columns represent methods. Values represent the ‐log10(p) of the Wilcoxon signed‐rank test between the accuracies of miDRW and the accuracies of other methods. The error bars represent standard deviation in (A), (B), and (C). [file MOL2-13-2211-s002.tif]

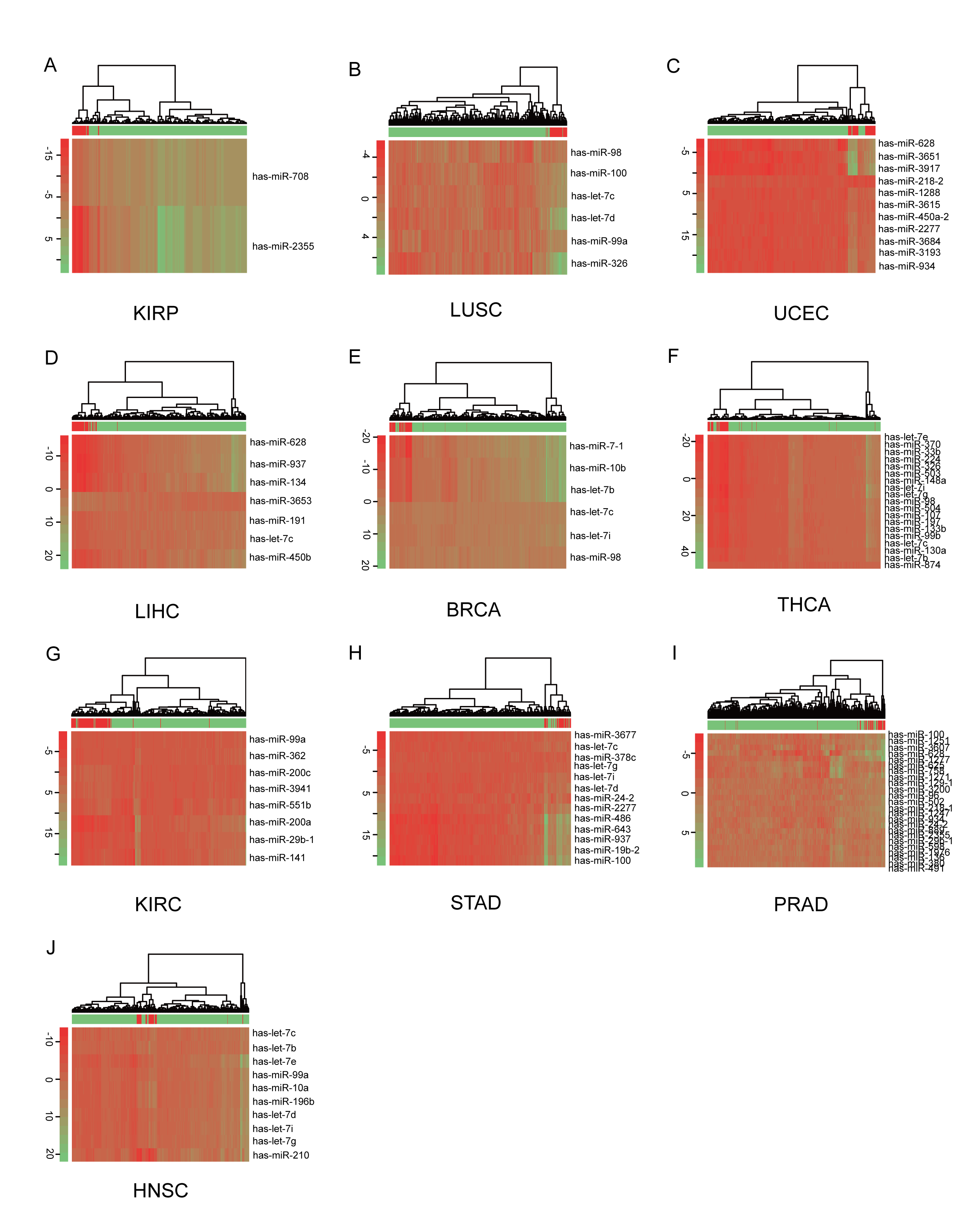

Supplement: Supplementary file 3 — Fig. S3. The hierarchical cluster analysis based on active miRNA‐mediated subpathways of other cancers before the median frequency. The row and column represent miRNA‐mediated subpathway and samples (the red and green bars represent normal and cancer samples), respectively. [file MOL2-13-2211-s003.tif]

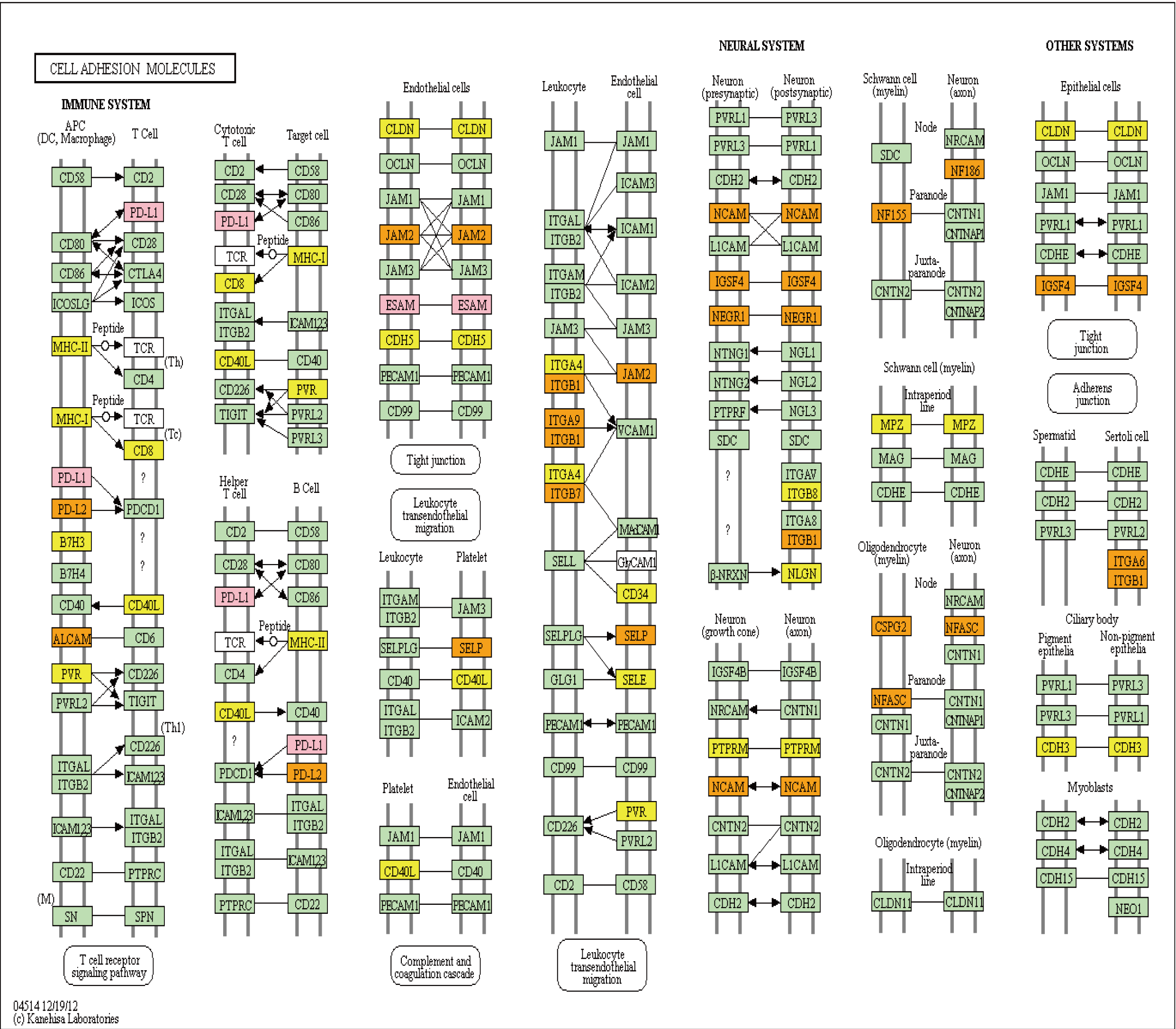

Supplement: Supplementary file 4 — Fig. S4. A snapshot of the Cell adhesion molecules (CAMs, hsa04514). The orange (yellow) color nodes represent the differentially expressed target genes of hsa‐miR‐134 (hsa‐miR‐326). The pink color nodes represent the common differentially expressed target genes of hsa‐miR‐134 and hsa‐miR‐326. [file MOL2-13-2211-s004.tif]

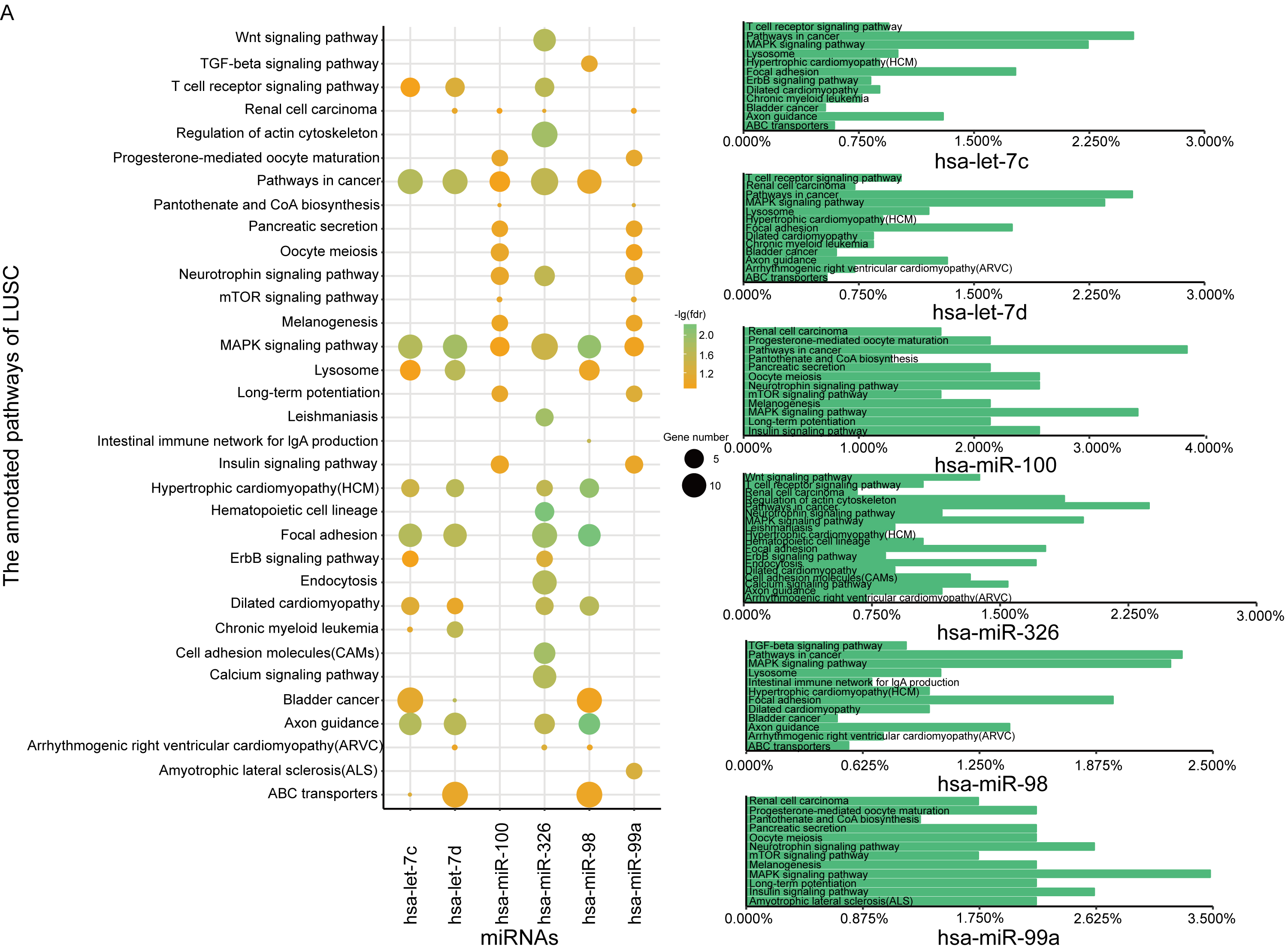

Supplement: Supplementary file 5 — Fig. S5. The summary bubble‐bar plot shows the functional enrichment results of the active miRNA‐mediated subpathways of other cancers. The bars on the right show the percentage of significantly differentially expressed genes annotated to the KEGG pathways. The bubble size indicates the number of genes in each KEGG pathway, and different colors correspond to different FDRs. The darker color indicates the smaller FDR. [file MOL2-13-2211-s005.tif]

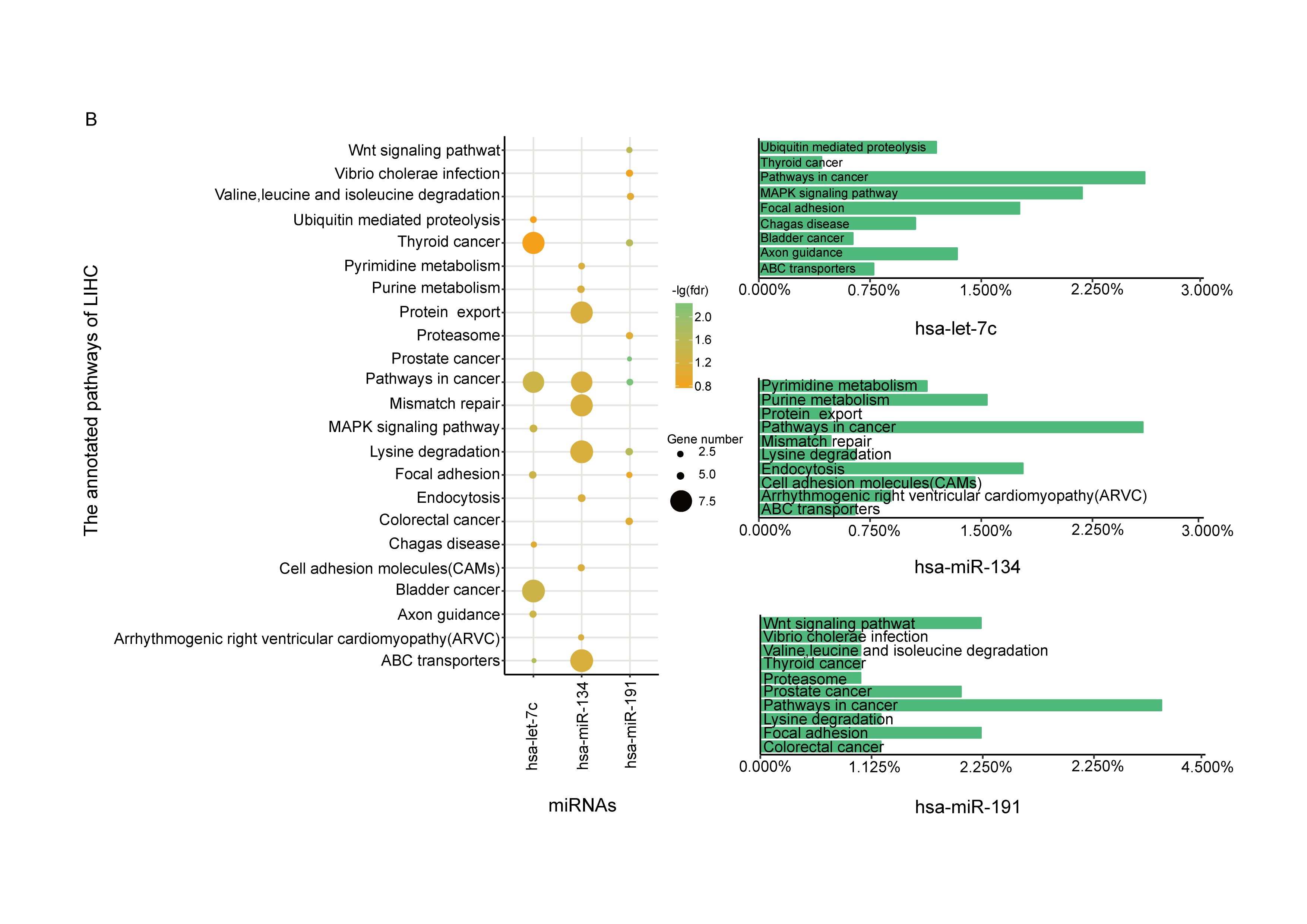

Supplement: Supplementary file 6 [file MOL2-13-2211-s006.tif]

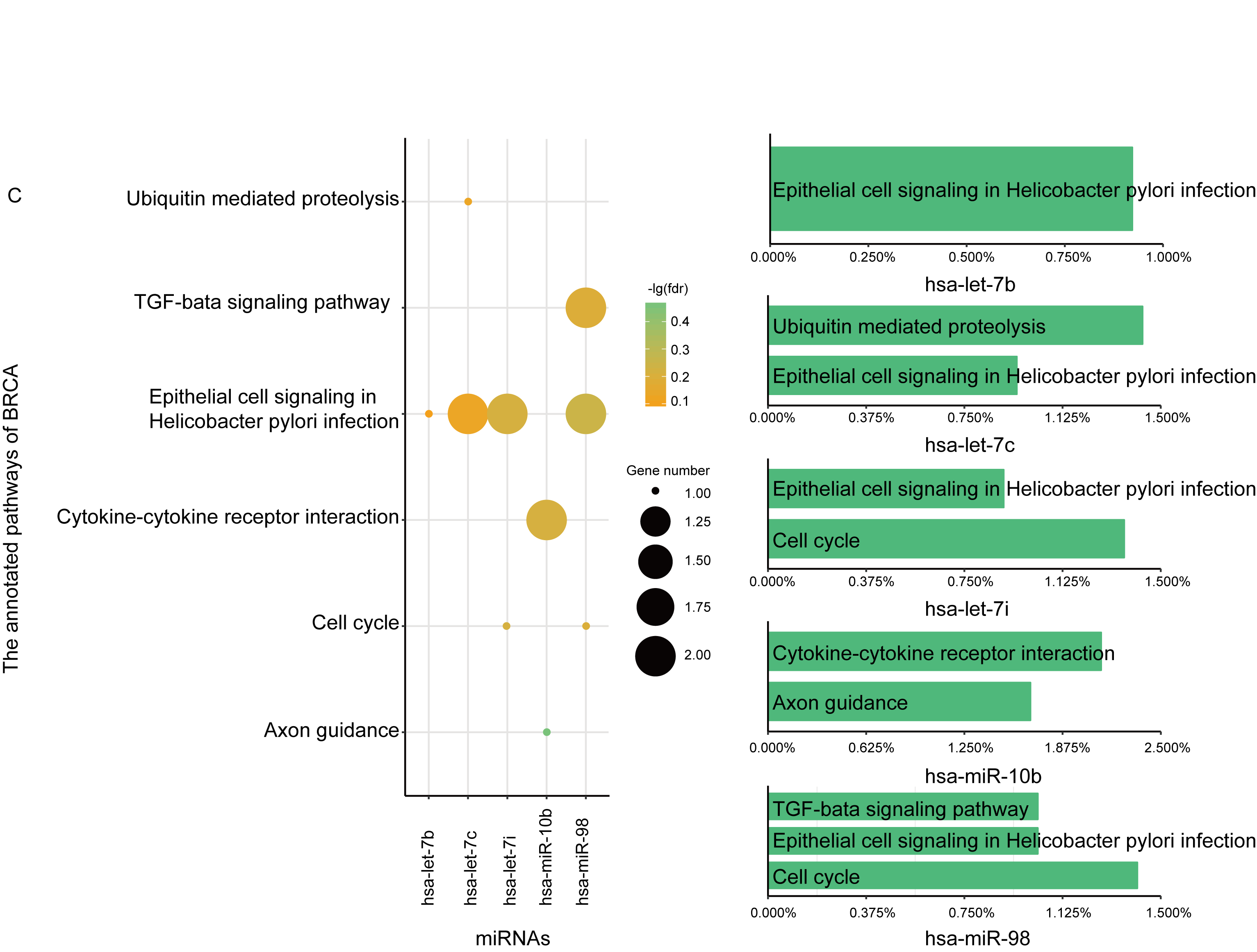

Supplement: Supplementary file 7 [file MOL2-13-2211-s007.tif]

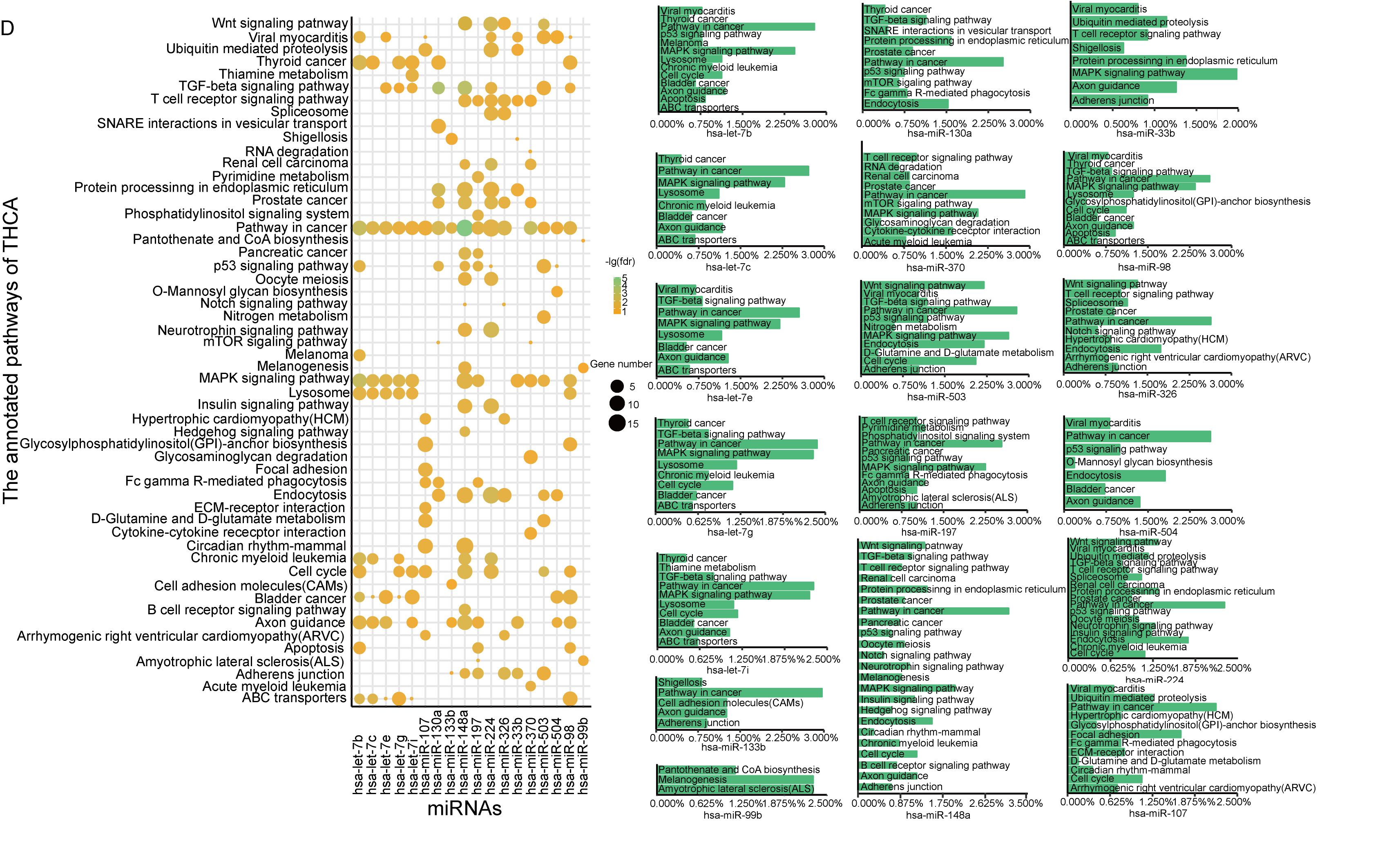

Supplement: Supplementary file 8 [file MOL2-13-2211-s008.tif]

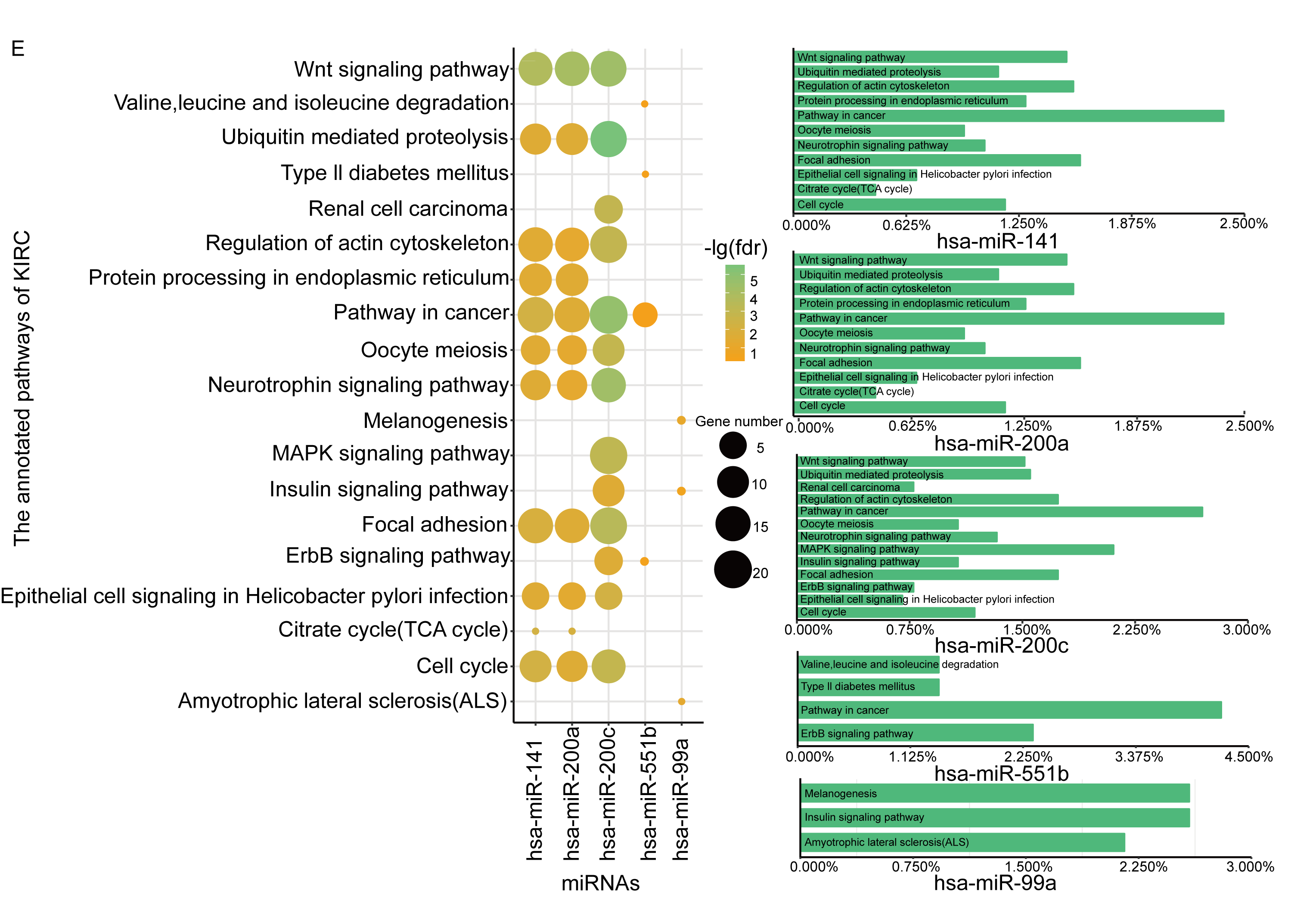

Supplement: Supplementary file 9 [file MOL2-13-2211-s009.tif]

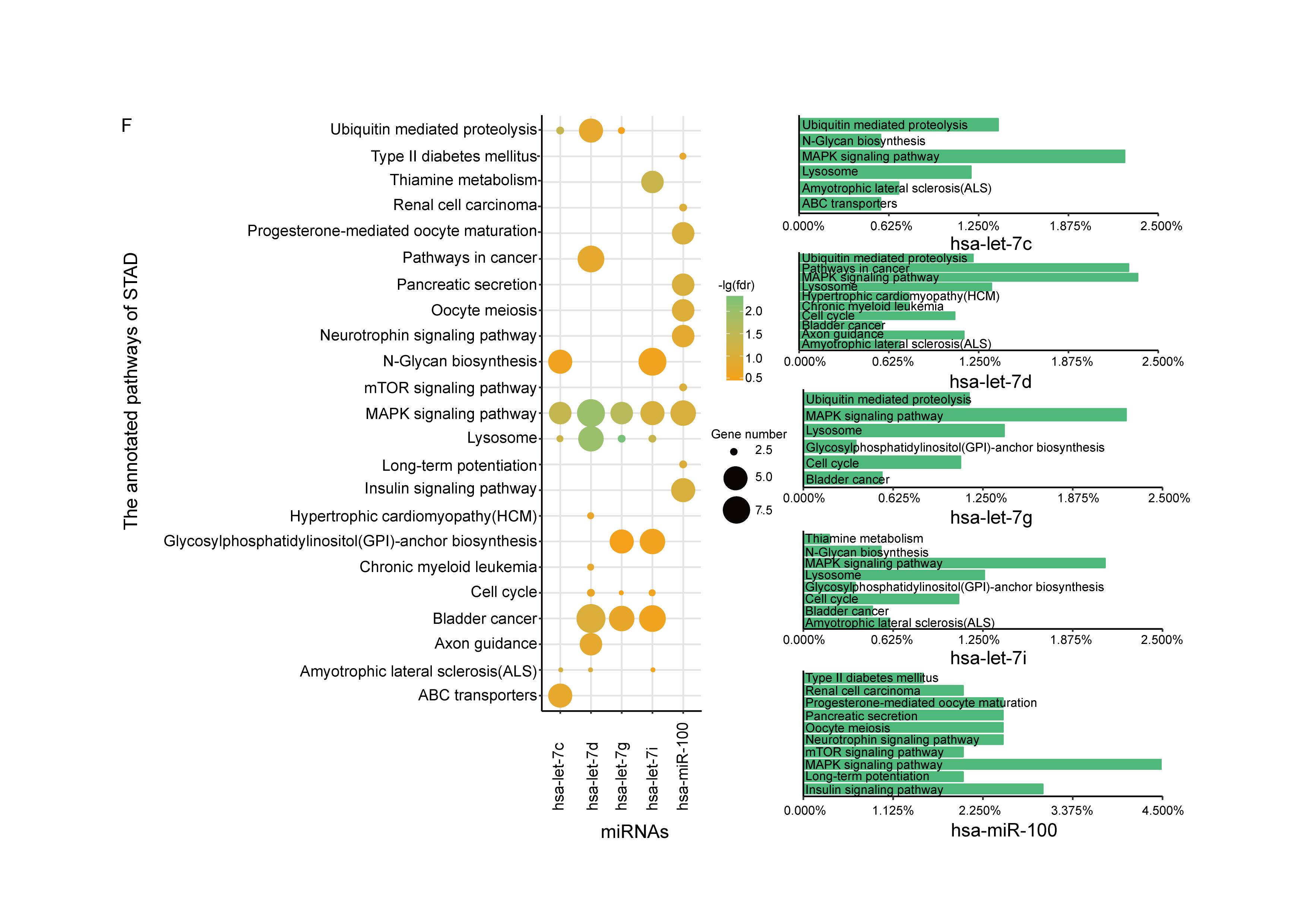

Supplement: Supplementary file 10 [file MOL2-13-2211-s010.tif]

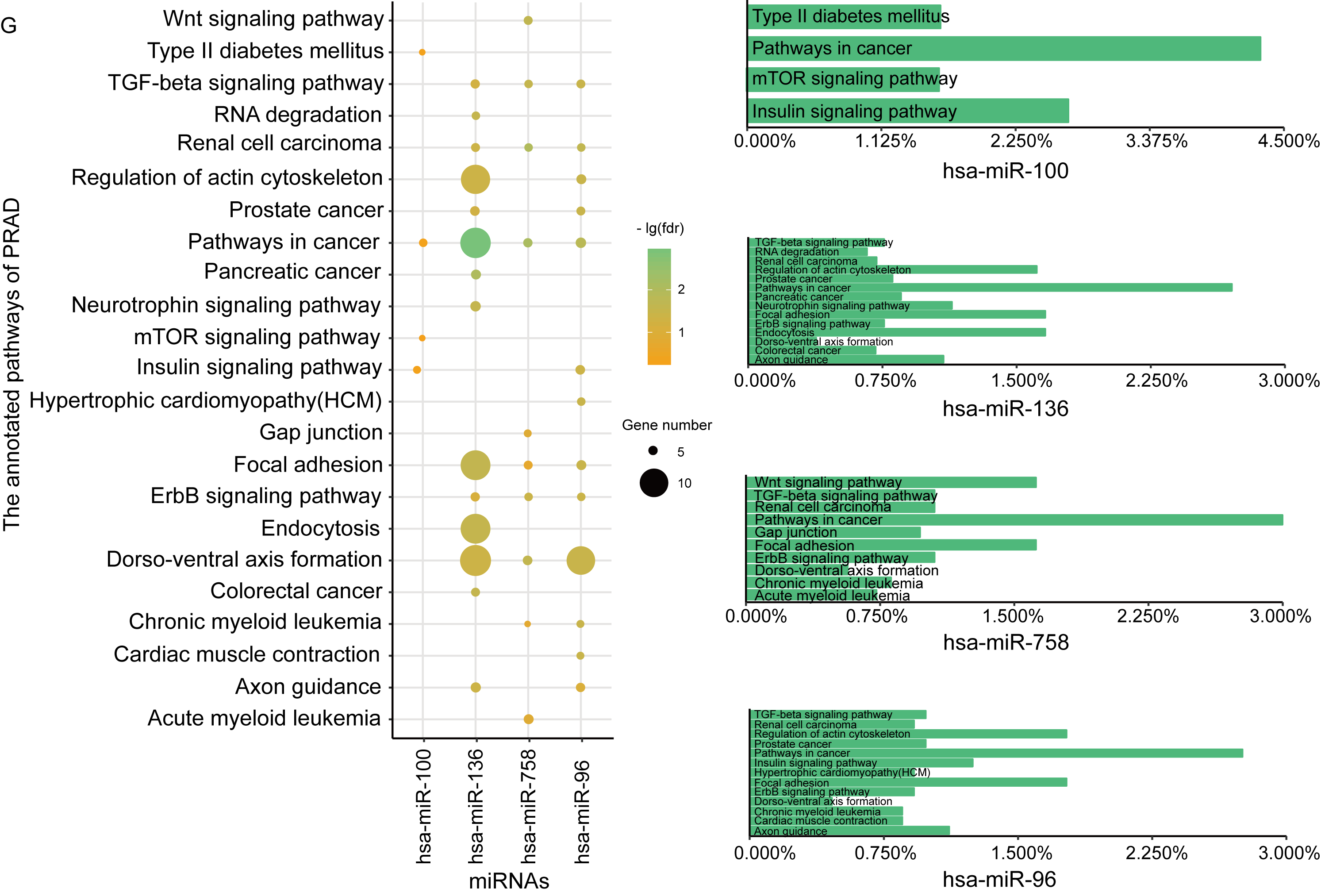

Supplement: Supplementary file 11 [file MOL2-13-2211-s011.tif]

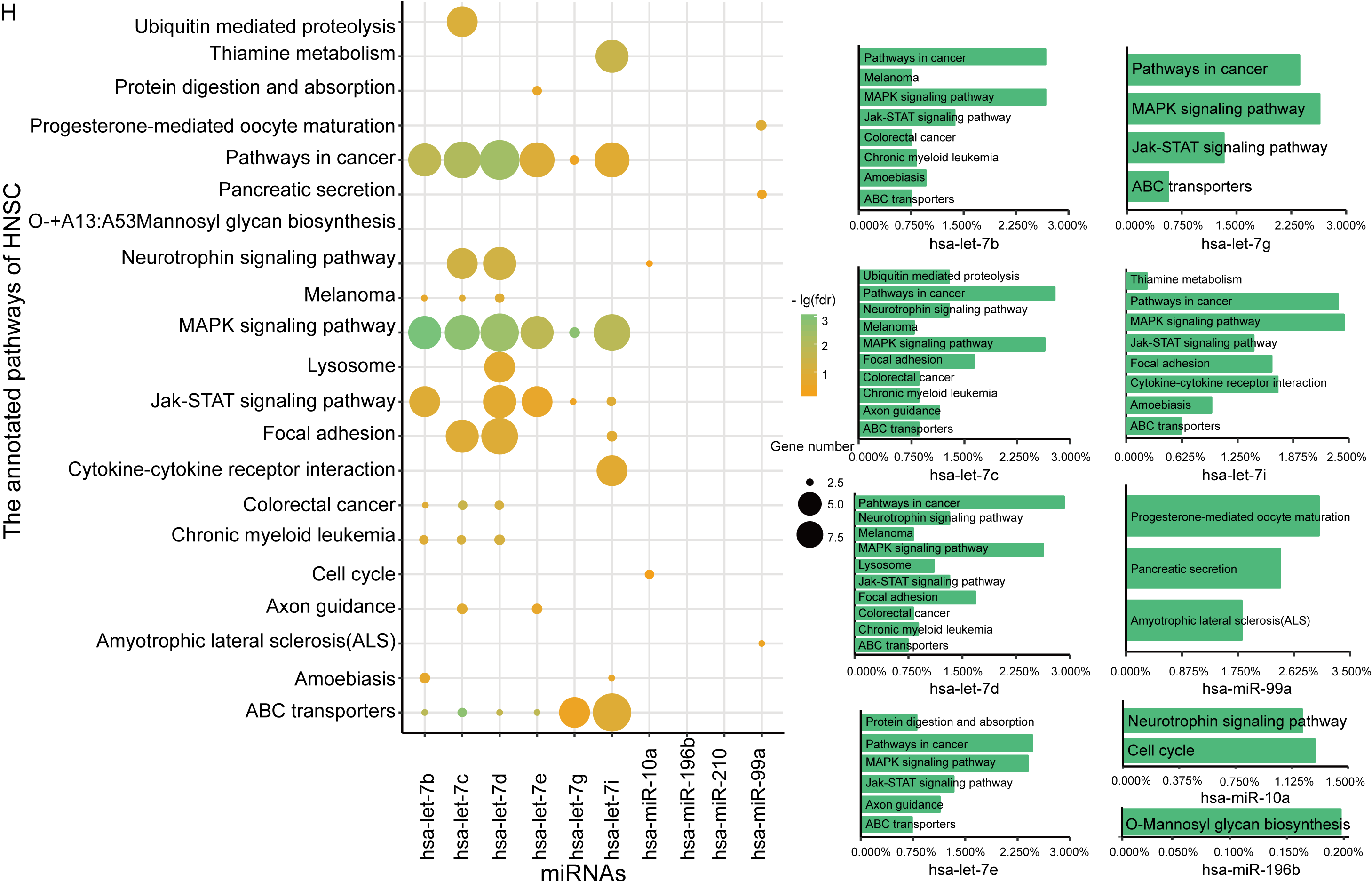

Supplement: Supplementary file 12 [file MOL2-13-2211-s012.tif]

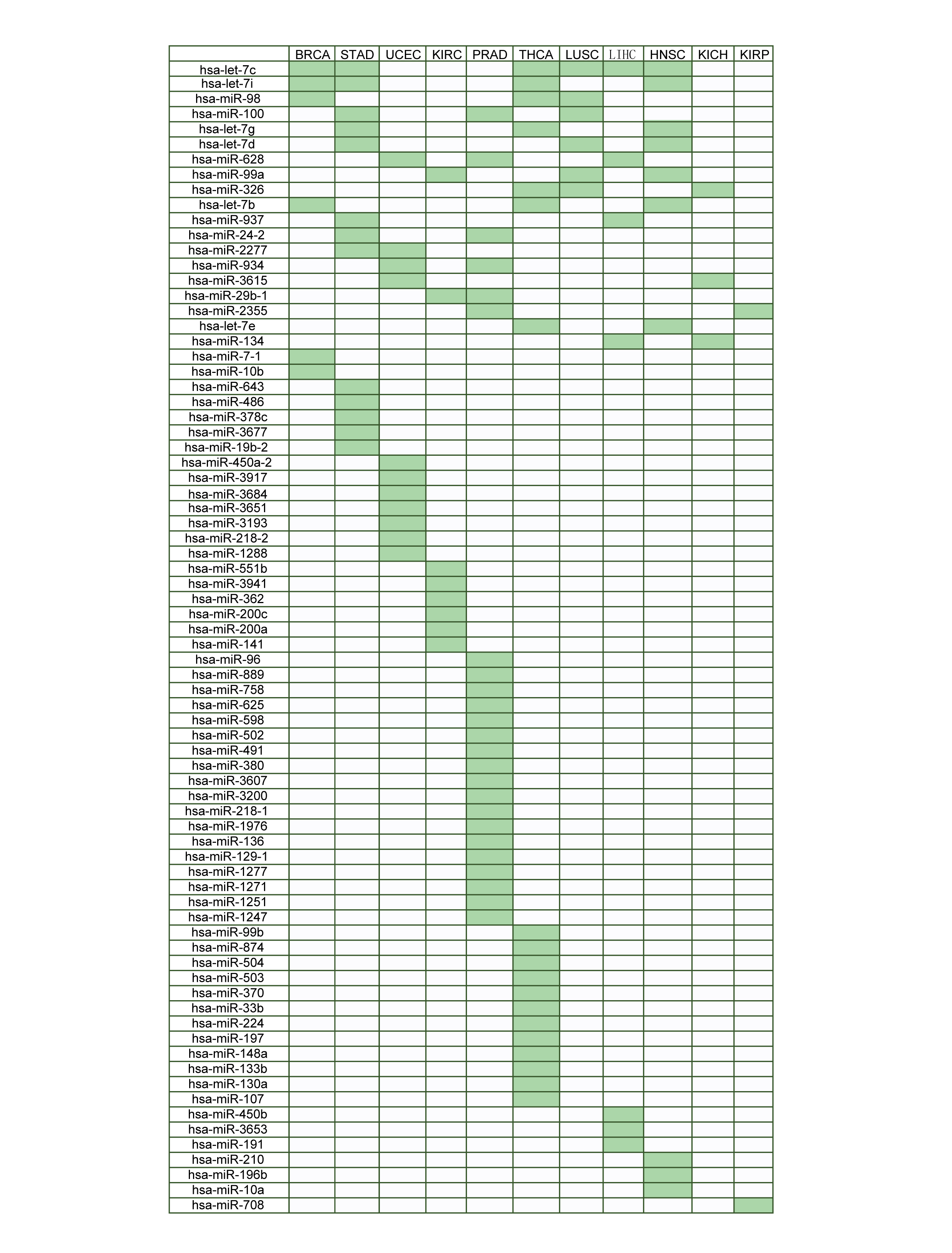

Supplement: Supplementary file 13 — Fig. S6. A global view of topologically inferring active subpathways and cancers. Each column represents one cancer, and each row represents an active miRNA‐mediated subpathway. [file MOL2-13-2211-s013.tif]

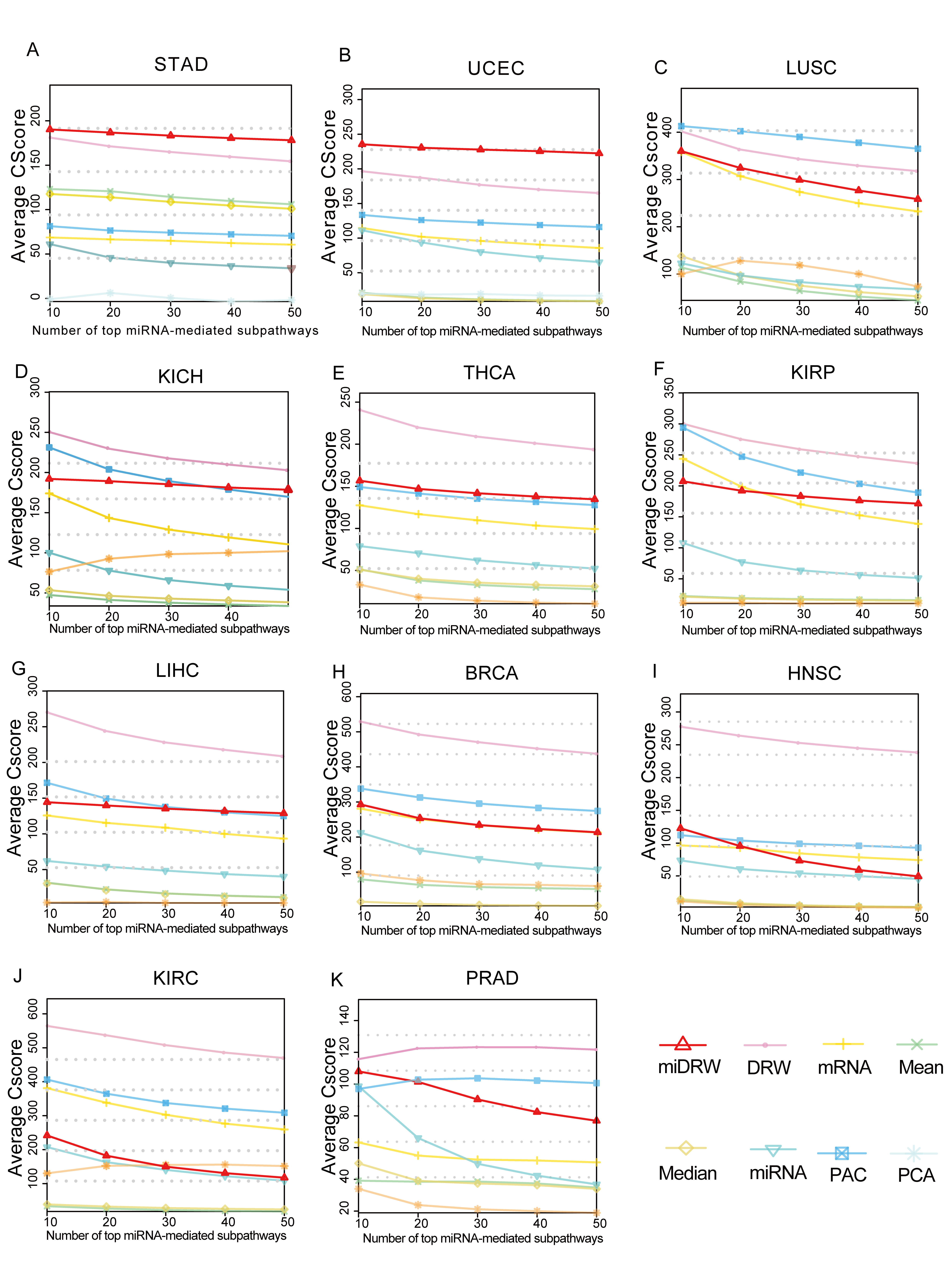

Supplement: Supplementary file 14 — Fig. S7. Reproducibility power of the miDRW method for within‐datasets and cross‐dataset experiments. (A)‐(J) The line indicates the reproducibility power of the miDRW method for within‐dataset experiments. The x‐axis represents the number of top miRNA‐mediated subpathways, and the y‐axis shows the reproducibility power Cscore of the top k miRNA‐mediated subpathways, k=10, 20, 30, 40. (K) The line indicates the reproducibility power of the miDRW method for PRAD cross‐dataset experiments. The x‐axis represents the number of top miRNA‐mediated subpathways, and the y‐axis shows the reproducibility power Cscore of the top k miRNA‐mediated subpathways, k=10, 20, 30, 40. [file MOL2-13-2211-s014.tif]
